# Transcriptomic and metabolomic analyses provide insight into the volatile compounds of citrus leaves and flowers

**DOI:** 10.1186/s12870-019-2222-z

**Published:** 2020-01-06

**Authors:** Haipeng Zhang, Mengjun Chen, Huan Wen, Zhenhua Wang, Jiajing Chen, Liu Fang, Hongyan Zhang, Zongzhou Xie, Dong Jiang, Yunjiang Cheng, Juan Xu

**Affiliations:** 1 Key Laboratory of Horticultural Plant Biology (Ministry of Education), College of Horticulture and Forestry, Huazhong Agricultural University, Wuhan, 430070 People’s Republic of China; 2 Citrus Research Institute of Southwest University, National Citrus Germplasm Repository, Chongqing, 400712 People’s Republic of China

**Keywords:** Citrus, Volatile profile, Terpenoid biosynthesis, Wild or semiwild germplasms, *STPS*

## Abstract

**Background:**

Previous reports have mainly focused on the volatiles in citrus fruits, and there have been few reports about the volatiles in citrus leaves and flowers. However, citrus leaves and flowers are also rich in volatile compounds with unique aromas. Here, to investigate the volatiles in citrus leaves and flowers, volatile profiling was performed on leaves from 62 germplasms and flowers from 25 germplasms.

**Results:**

In total, 196 and 82 volatile compounds were identified from leaves of 62 citrus germplasms and flowers of 25 citrus germplasms, respectively. The dominant volatile terpenoids were more diverse in citrus leaves than in peels. A total of 34 volatile terpenoids were commonly detected in the leaves of at least 20 germplasms, among which 31 were overaccumulated in the leaves of wild or semiwild germplasms. This result was consistent with the high expression levels of five genes and one key gene of the mevalonate and 2-C-methyl-D-erythritol-4-phosphate (MEP) biosynthetic pathways, respectively, as well as the low expression levels of geranylgeranyl diphosphate synthase of the MEP pathway, relative to the levels in cultivars. Fully open flowers showed increased levels of four terpene alcohols and a decrease in sabinene content compared with balloon-stage flowers, especially in sweet orange. A monoterpene synthase gene was identified and functionally characterized as a sabinene synthase in vitro.

**Conclusions:**

Collectively, our results suggest that 31 important terpenoids are abundant in wild or semiwild citrus germplasms, possibly because of a negative effect of domestication on the volatiles in citrus leaves. The sweet smell of fully open flowers may be attributed to increased levels of four terpene alcohols. In addition, a sabinene synthase gene was identified by combined transcriptomic and metabolomic analyses.

## Background

Thousands of metabolites in plants, such as flavonoids [1], carotenoids, limonoids, nomilins [2], furanocoumarins [3] and volatile terpenoids [4], have various functions with biological effects that are beneficial to humans [5]. The 2-C-methyl-D-erythritol-4-phosphate (MEP) and mevalonate (MVA) pathways in the plastids and cytoplasm of plants, respectively, synthesize C5 isopentenyl pyrophosphate (IPP) [5, 6]. IPP and its isomer dimethylallyl diphosphate (DMAPP) are substrates for the biosynthesis of geranyl pyrophosphate (GPP) and farnesyl pyrophosphate (FPP) in plastids and the cytoplasm, respectively [6, 7]. Monoterpenes (C_10_) are synthesized from GPP and sesquiterpenes (C_15_) are synthesized from FPP by various terpene synthesis (*TPS*) genes [8]. The monoterpenes and sesquiterpenes may then be utilized to synthesize various terpene derivatives by cytochrome P450 (CYP450) enzymes, dehydrogenases and reductases [7, 9]. For example, CYP76C1 and CYP76C3 metabolize linalool to 8-hydroxylinalool in *Arabidopsis* [9, 10].

As one of the most important groups of plant volatiles, terpenoids are widely present in higher plants and participate in a wide range of biological activities [5]. Terpenoids are primary metabolites or secondary metabolites and play important roles in plant-microorganism, plant-insect, plant-animal and plant-plant interactions [11, 12]. The terpenes accumulated in plants not only participate in direct or indirect defense against insects or bacteria [11–13] but also contribute to specific aromas that benefit the plants. Moreover, volatile terpenoids are widely used as important components in essential oils or as precursors for the synthesis of flavor products. For example, sabinene has anti-inflammatory activity [14]; *β*-linalool, which has an anti-inflammatory effect, is a widely used food and perfume additive [15]; *β*-myrcene is often used as raw material for the synthesis of *β*-linalool and geraniol [16]; linalyl acetate has been reported to possess anti-inflammatory activity [15]; and *β*-elemene has antitumor activity [17].

Terpenoids are mainly produced by various *TPS* genes in plants. Previous studies have indicated that there are 95 *TPS* loci in the genome of Valencia sweet orange [18], few of which have been functionally characterized. *CsTPS1* has been demonstrated to function in the production of (+)-valencene in sweet orange [19]. *CitMTSE1, CitMTS3, CitMTS61* and *CitMTS62* are associated with the production of *D*-limonene, γ-terpinene, γ-terpinene and *β*-pinene, respectively, in *C. unshiu* [20]; *Cs3g04360.1* was found to affect the production of *D*-limonene in Newhall navel orange [21]. In addition, seven sesquiterpene synthase genes have been characterized in sweet orange [18]. Due to the high sequence similarities of *TPS* genes, it is difficult to explore candidate genes based on sequence homology and genomic annotation. Therefore, a combination of transcriptomic and metabolomic analyses may be a good approach to narrow the range of candidate genes.

As some of the most important fruit crops worldwide, citrus plants are rich in volatile terpenoids [22]. Notably, studies focusing on volatile compounds of citrus plants can be dated back to 1925 [23]. Currently, citrus flavor is one of the most important flavors in the essential-oil industry and is used in 25% of essential oils [24]. Various citrus essential oils, such as sweet-orange oils, lemon oils and grapefruit oils, are extracted from citrus fruits, flowers and leaves [24]. Overall, there is abundant evidence showing the passionate pursuit of citrus volatiles by humans.

Citrus plants include several hundreds of germplasms, with the Swingle [25] and Tanaka [26] systems being the most widely accepted systems for germplasm classification [22, 27, 28]. Recently, metabolomic analysis, especially determination of volatile profiles, has been used in the classification of citrus plants from a chemotaxonomic perspective [22, 27, 28]. Previous studies have shown the chemotaxonomy of *Citrus*, *Poncirus* and *Fortunella* using principal component analysis (PCA) and hierarchical cluster analysis (HCA) based on peel and leaf volatiles and have discussed the origin of Mangshanyegan (*Citrus nobilis* Lauriro) [28]. Classification of 20 citrus germplasms was carried out based on their fruit peel volatiles using HCA and partial least squares discriminant analysis (PLS-DA) [27]. Zhang et al. [22] analyzed the relationships among different citrus species through HCA based on volatiles from the peels of 108 citrus germplasms. Constrained principal coordinate analysis (CPCoA) is always used to determine the differences in volatile compounds among different species. The above classifications are similar to those of the existing Swingle system, demonstrating the reliability of the chemotaxonomy based on the volatile profiles of various citrus genotypes.

Recent studies and our previous reports have been mainly focused on volatiles in citrus fruits [4, 22, 24, 29, 30], while the volatiles in leaves and flowers are less frequently reported. However, citrus leaves and flowers are also rich in volatile compounds and can emit specific volatiles upon insect attack to attract natural enemies to prey on the insects [11] or to defend against fungal pathogens [12]. Citrus leaves, which are characterized by a short growth period, high biomass and availability throughout the year, and citrus flowers, which emit specific volatiles at different opening stages, are both highly valuable for the citrus essential-oil industry, for the studies of genotype- and spatiotemporal-specific gene expression and regulation, and for elucidation of the interaction mechanisms among plants, animals, microorganisms and insects.

This study aims to identify and better understand the metabolism of volatiles, then explore the candidate genes governing the biosynthesis of various volatile compounds with special biological benefits for plants and humans in citrus leaves and flowers. The volatile profiles of leaves and flowers were determined. Furthermore, transcriptome analyses and RT-qPCR were used to examine the domestication of volatiles in citrus leaves. The transcriptome data and volatile profiling data from two different opening stages of Cara Cara navel orange were subjected to correlation analysis to identify candidate *TPS*s and *CYP450*s. Furthermore, the mechanism underlying the differentiation of volatile profiles between cultivated and wild or semiwild citrus plants and the physiological data for leaf and flower volatiles were analyzed for optimal utilization of various citrus germplasms in breeding or in the essential-oil industry.

## Results

### Volatile compounds detected in Citrus leaves

A total of 196 volatile compounds (Additional file 1: Table S1) were tentatively detected in the leaves of 62 citrus germplasms (Table 1) based on the NIST Mass Spectral Library, among which 57 compounds were identified with authentic standards (Additional file 2: Table S2). The 196 compounds could be classified into 16 groups, including 19 monoterpenes, 15 monoterpene alcohols, five monoterpene aldehydes, five monoterpene ketones, five monoterpene oxides, six monoterpene esters, 72 sesquiterpenes, 16 sesquiterpene alcohols, one sesquiterpene oxide, two sesquiterpene aldehydes, five alcohols, 15 aldehydes, five acids, five esters, four ketones and 16 other compounds (Additional file 1: Table S1).

**Table 1:** Leaves of citrus germplasms investigated in this study. (Cited firstly in line 150)

No	Germplasms	Scientific names	Abb	Production area	Total Content (mg/g, FW)	Total Compounds
1	Nongxianghonghedayicheng	*C. hystrix*	NXH	RL^1^	15.52 ± 0.84	49
2	Baihuahonghedayicheng	*C. hystrix*	BHH	RL^1^	12.26 ± 0.48	61
3	Zihuahonghedayicheng	*C. hystrix*	ZHH	RL^1^	12.53 ± 0.65	64
4	Honghedayicheng	*C. hystrix*	HHD	BB^2^	14.42 ± 3.21	61
5	Ichangensis ‘No.4’	*C. ichangensis*	IC4	BB^2^	3.39 ± 0.36	57
6	Ichangensis ‘2583’	*C. ichangensis*	IC2	BB^2^	2.59 ± 1.23	49
7	Ichangensis ‘6–3’	*C. ichangensis*	IC6	BB^2^	6.14 ± 0.75	57
8	Ichangensis ‘huaihua’	*C. ichangensis*	ICH	BB^2^	4.75 ± 0.23	49
9	Ichangensis	*C. ichangensis*	IC	BB^2^	3.16 ± 0.10	59
10	Ichangensis ‘chuihua’	*C. ichangensis*	ICC	BB^2^	2.09 ± 0.37	48
11	Ichangensis ‘baihua’	*C. ichangensis*	ICB	BB^2^	4.90 ± 0.55	55
12	Ziyangxiangcheng	*C. junos*	ZYX	BB^2^	5.19 ± 0.55	47
13	Guanxianxiangcheng No.3	*C. junos*	GX3	BB^2^	5.87 ± 0.41	39
14	Qianjiangxiangcheng No.3	*C. junos*	QJ3	BB^2^	5.80 ± 0.43	46
15	Wanmucheng	*C. aurantium*	WMC	BB^2^	3.14 ± 0.40	50
16	Morocco sour orange	*C. aurantium*	MSO	BB^2^	4.13 ± 0.30	32
17	Brazil sour orange	*C. aurantium*	BSO	BB^2^	2.07 ± 0.17	29
18	Daidai sour orange	*C. aurantium*	DDS	BB^2^	2.76 ± 0.14	19
19	Defuniya sour orange	*C. aurantium*	DSO	BB^2^	11.05 ± 0.68	41
20	Goutou cheng	*C. aurantium*	GTC	BB^2^	6.96 ± 1.03	64
21	Xingshan sour orange	*C. aurantium*	XSO	BB^2^	0.13 ± 0.01	28
22	Xiaoye sour orange	*C. aurantium*	XYS	BB^2^	4.59 ± 1.07	32
23	Nanchuan citron	*C. medica*	NCC	BB^2^	4.79 ± 0.20	45
24	Muli citron	*C. medica*	MLC	BB^2^	7.81 ± 0.65	49
25	Citron	*C. medica*	CY	BB^2^	2.88 ± 0.40	53
26	Yuanjiang citron	*C. medica*	YJC	BB^2^	4.03 ± 0.44	42
27	Danna citron	*C. medica*	DNC	BB^2^	6.32 ± 0.74	41
28	Finger citron	*C. medica*	FGC	BB^2^	7.09 ± 3.00	48
29	Red limonia	*C. medica*	RL	BB^2^	4.67 ± 0.61	57
30	Limonia	*C. medica*	LIM	BB^2^	15.52 ± 0.84	62
31	Lime	*C. medica*	LIE	BB^2^	12.26 ± 0.48	65
32	Newhall navel orange	*C. sinensis*	NNO	WH^3^	3.99 ± 0.34	56
33	Seika navel orange	*C. sinensis*	SNO	WH^3^	2.80 ± 0.14	53
34	Lane late navel orange	*C. sinensis*	LNO	WH^3^	4.12 ± 0.11	59
35	Chandler pummelo	*C. maxima*	CHP	BB^2^	0.82 ± 0.08	23
36	Ni 800 pummelo	*C. maxima*	N800	BB^2^	1.62 ± 0.09	24
37	Shatian pummelo	*C. maxima*	STP	GZ^4^	2.15 ± 0.12	44
38	Thai pummelo	*C. maxima*	TP	WH^3^	1.49 ± 0.21	33
39	Wanbai pummelo	*C. maxima*	WBP	WH^3^	0.53 ± 0.01	24
40	low-acid pummelo	*C. maxima*	LAP	WH^3^	1.45 ± 0.10	37
41	Zipi pummelo	*C. maxima*	ZPP	WH^3^	1.87 ± 0.12	31
42	Fenghuang pummelo	*C. maxima*	FHP	WH^3^	1.23 ± 0.44	23
43	Kaopan pummelo	*C. maxima*	KPP	WH^3^	0.22 ± 0.03	34
44	HB pummelo	*C. maxima*	HBP	WH^3^	1.90 ± 0.26	13
45	Huanong red pummelo	*C. maxima*	HNP	WH^3^	1.34 ± 0.10	36
46	Star ruby grapefruit	*C. paradisi*	SRG	WH^3^	1.62 ± 0.09	31
47	Niedu wild tangerine	*C. reticulata*	NDJ	BB^2^	16.2 ± 1.25	52
48	Tuju tangerine	*C. reticulata*	TJ	BB^2^	9.79 ± 0.67	53
49	Guoqing No.1	*C. unshiu*	GQ1	WH^3^	2.39 ± 0.33	36
50	Mangshanyegan	*C. mangshanensis*	MSY	WH^3^	2.87 ± 0.45	39
51	Shatang tangerine	*C. reticulata*	STJ	GZ^4^	7.53 ± 1.84	28
52	Red tangerine	*C. reticulata*	RT	WH^3^	8.04 ± 4.00	46
53	Yaoxianggan	*C. reticulata*	YXG	BB^2^	28.69 ± 1.94	69
54	India sour tangerine	*C. reshni*	IST	BB^2^	11.10 ± 1.08	58
55	India wild tangerine	*C. indica*	IWT	BB^2^	4.92 ± 1.05	52
56	Chachi	*C. reticulata*	CZG	GZ^4^	12.66 ± 1.00	43
57	Cupigoushigan	*C. reticulata*	CPG	BB^2^	13.07 ± 0.77	50
58	Damaliu	*C. reticulata*	DML	BB^2^	10.78 ± 0.56	62
59	Mangshan tangerine	*C. reticulata*	MST	BB^2^	9.99 ± 2.32	63
60	Wulong sour orange	*C. reticulata*	WLS	BB^2^	9.21 ± 0.23	55
61	Xipigoushigan	*C. reticulata*	XPG	BB^2^	9.16 ± 0.80	54
62	*Poncirus trifoliata*	*Poncirus trifoliata*	TRI	WH^3^	12.53 ± 0.65	41

*RL*^*1*^ Ruili, Yunnan; *BB*^*2*^ Beibei, Chongqing; *WH*^*3*^ Wuhan, Hubei; *GZ*^*4*^ Guangzhou, Guangdong

The total volatile content (TVC) of different citrus germplasms ranged from 0.13 ± 0.01 mg/g (Xingshan sour orange) to 28.69 ± 1.94 mg/g (Yaoxianggan, YXG, loose-skin mandarin), and the number of compounds in each germplasm ranged from 13 (HB pummelo) to 69 (YXG) (Table 1).

There was higher diversity of the dominant compounds among different species in leaves than in peels. For example, the most abundant compounds were citronellal, geranyl acetate and *trans-β*-ocimene in papeda; *trans-β*-ocimene, linalyl acetate, α-pinene, (*E*)-*β*-farnesene and *γ*-elemene in *Citrus ichangensis*; sabinene, *γ*-terpinene, *D*-limonene and germacrene D in *Citrus junos*; *D*-limonene, α-citral, *β*-citral and citronellal in citron; *β*-pinene, (+)-bicyclogermacrene, *trans-β*-ocimene and caryophyllene in pummelo; linalool, *trans-β*-ocimene and *β*-elemene in loose-skin mandarin; sabinene, *trans-β*-ocimene and 3-carene in sweet orange; and linalyl acetate, linalool, caryophyllene and *β*-pinene in sour orange (Additional file 3: Table S3).

A total of 43 compounds were commonly detected in at least 20 germplasms, including 34 terpenoids. Notably, among these terpenoids, 31 were found at the highest levels in wild or semiwild germplasms. For example, eight terpenoids (sabinene, *β*-myrcene, *D*-limonene, *allo*-ocimene, α-elemene, *β*-elemene, (−)-humulene and germacrene D) were the most abundant in YXG; seven terpenoids (camphene, *β*-pinene, isospathulenol, germacrene D-4-ol, *trans-β*-ocimene, α-copaene and terpinolene) were at the highest levels in Niedu wild tangerine (NDT, loose-skin mandarin); *γ*-elemene and germacrene B were the most abundant in Indian sour tangerine (loose-skin mandarin); (+)-*δ*-cadinene and (+)-bicyclogermacrene were the most abundant in Wulong sour mandarin (loose-skin mandarin); and α-phellandrene and 3-carene were the most abundant in Muli citron (citron) and Newhall navel orange (sweet orange), respectively (Table 2).

**Table 2:** Thirty-four terpenoids abundant in citrus leaves of various germplasms. (Cited firstly in line 183)

Citrus germplasms	Special volatile compounds
Niedu wild mandarin^1^	camphene, *β*-pinene, isospathulenol, germacrene D-4-ol, *trans*-*β*-ocimene, *α*-copaene, terpinolene
Yaoxianggan^1^	sabinene, *β*-myrcene, *D*-limonene, allo-ocimene, *α*-elemene, *β*-elemene, (−)-humulene, germacrene D
Honghedayicheng^1^	*α*-citral, *β*-citral, citronellal, geranyl acetate
Cupigoushigan^1^	*α*-pinene, linalool
India sour tangerine^1^	*γ*-elemene, germacrene B
Wulong sour orange^1^	(+)-bicyclogermacrene, (+)-*δ*-cadinene
Mangshan tangerine^1^	nerolidol, *α*-thujene
Limonia^1^	*α*-terpineol
Nanchuan citron^1^	nerol acetate
Xipigoushigan^1^	*δ*-elemene
*Poncirus trifoliata* ^1^	caryophyllene
Muli citron^2^	*α*-phellandrene
Newhall navel orange^2^	3-carene
Fingered citron^2^	*cis*-*β*-ocimene

^1^: wild or semi-wild germplasms; ^2^:cultivars

**Fig. 1:**
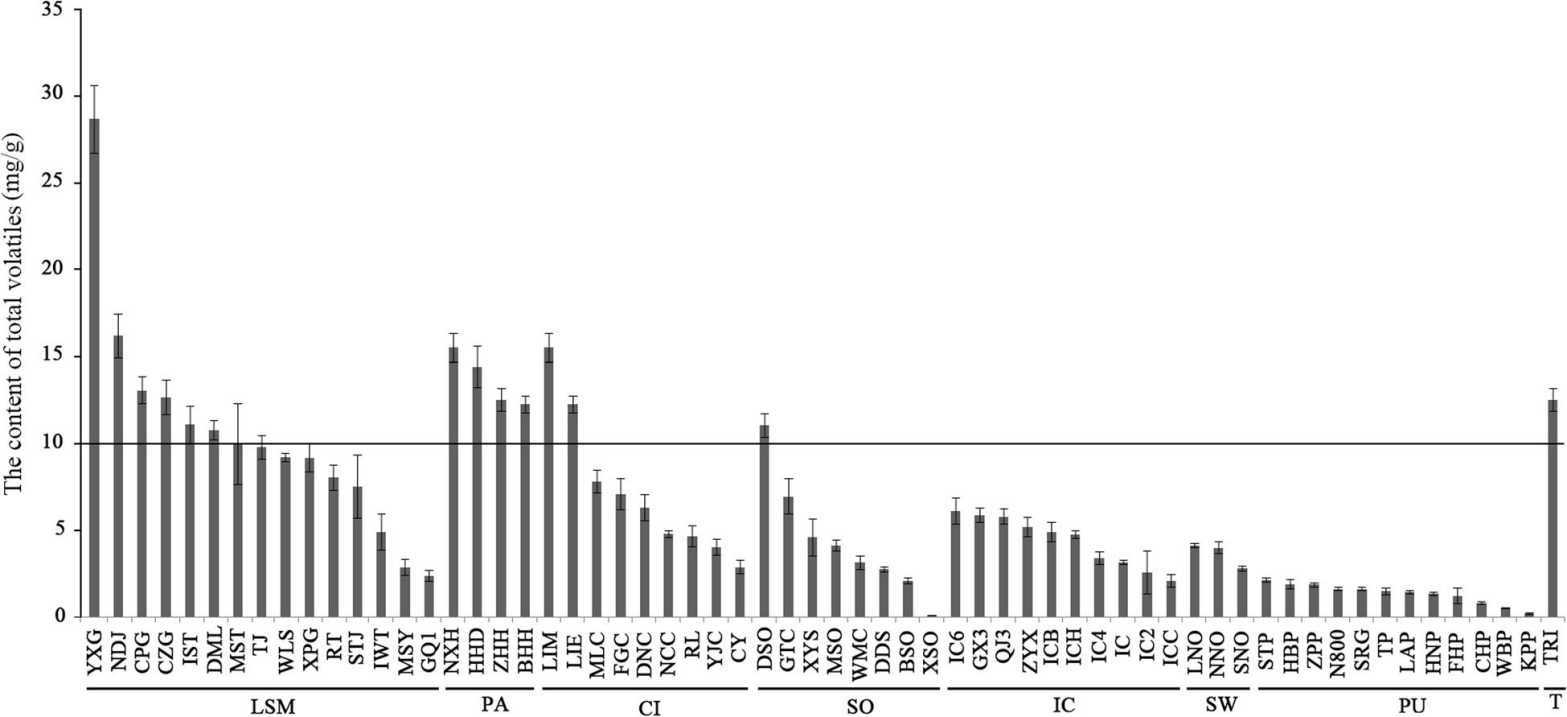
Total volatile content in citrus leaves. The line above shows the 14 germplasms with the highest total volatile levels among all 62 citrus germplasms. LSM: loose-skin mandarin; PA: papeda; CI: citron; SO: sour orange; IC: *C. ichangensis*; SW: sweet orange; PU: pummelo; TRI: *Poncirus trifoliata*

All 62 investigated germplasms could be classified into seven species. The TVC level was > 10 mg/g for most papeda and loose-skin mandarin germplasms; 3–10 mg/g for most *C. ichangensis* germplasms, citron, sweet orange and sour orange germplasms; and < 2.5 mg/g for most pummelo germplasms (Fig.1). Among all seven citrus species, loose-skin mandarin and sweet orange had the largest and smallest numbers of volatile compounds, respectively.

A total of 140 volatile compounds were detected in the leaves of 15 loose-skin mandarin germplasms. The total content varied widely from 2.39 ± 0.33 mg/g in Guoqing No.1 (GQ.1) to 28.69 ± 1.94 mg/g in YXG, and more than 50 compounds were identified in most loose-skin mandarin germplasms (Table 1). Monoterpenes accounted for 42–78% of the TVC in loose-skin mandarin, except in Chachi and red-tangerine (Additional file 4: Figure S1); Chachi and red-tangerine leaves specifically accumulated methyl anthranilate, which accounted for 69.88 and 43% of the TVC, respectively, while *D*-limonene, linalool, and sabinene were dominant volatile compounds in other loose-skin mandarin germplasms. Sesquiterpenoids only accounted for 5% of the TVC in Chachi, which was significantly lower than that in other mandarin germplasms (Additional file 1: Table S1 and Additional file 5: Figure S2).

In 10 *C. ichangensis* germplasms, 128 volatile compounds were detected, including 76 sesquiterpenoids, which accounted for 30–50% of the TVC (Table 1; Additional file 4: Figure S1). In *C. junos*, 55 compounds were identified, including 47 terpenoids. Among these volatile compounds, 73 and 13 compounds were unique in *C. ichangensis* and *C. junos*, respectively (Additional file 1: Table S1). Although the number of compounds detected in *C. junos* was lower than that detected in *C. ichangensis*, *C. junos* exhibited a higher TVC level. Notably, sabinene, the levels of *β*-cubebene, germacrene D and *β*-elemene in *C. junos* were significantly higher than those in most *C. ichangensis* germplasms (> 10-fold),while *trans-β*-ocimene and (+)-*δ*-cadinene were present at significantly higher levels in *C. ichangensis* than in *C. junos*(> 10-fold) (Additional file 1: Table S1).

In the leaves from 9 citron germplasms, 119 volatile compounds were identified, with TVC ranging from 4.03 ± 0.44 mg/g (Yuanjiang citron) to 15.52 ± 0.84 mg/g (red-limonia, RL) and number of compounds ranging from 41 (Danna citron) to 65 (lime) (Table 1). Monoterpenes were the dominant compounds in citron germplasms (Additional file 4: Figure S1). RL leaves contained 60 volatile compounds and had the highest TVC, with *D*-limonene being the most abundant, followed by *β*-pinene, (*Z, Z*)-α-farnesene, and caryophyllene (Additional file 1: Table S1).

In 12 pummelo germplasms, 73 volatile compounds were detected. The TVC ranged from 0.22 ± 0.03 mg/g (Kaopan pummelo) to 2.15 ± 0.12 mg/g (Shatian pummelo) (Table 1), with monoterpenes being the most abundant in most pummelo germplasms and accounting for more than 50% of the TVC (Additional file 4: Figure S1). The TVC of HB pummelo leaves was low (1.90 ± 0.26 mg/g), and sesquiterpenes were almost undetectable (Additional file 1: Table S1).

In eight sour-orange and three sweet-orange germplasms, 88 and 64 volatile compounds were detected, respectively. Monoterpenes were the most abundant in both orange species (Additional file 4: Figure S1). As many as 52 compounds were detected in the leaves of Goutou Cheng (Table 1), with *β*-pinene being the most abundant, followed by linalool, caryophyllene, sabinene and *trans*-*β*-ocimene. The most abundant compounds were linalyl acetate in Xiaoye sour orange; linalool and (+)-bicyclogermacrene in Defuniya sour orange; and *trans*-*β*-ocimene and sabinene in sweet orange (Additional file 1: Table S1 and Additional file 3: Table S3).

### CPCoA of Citrus species based on leaf volatile profiles

**Fig. 2:**
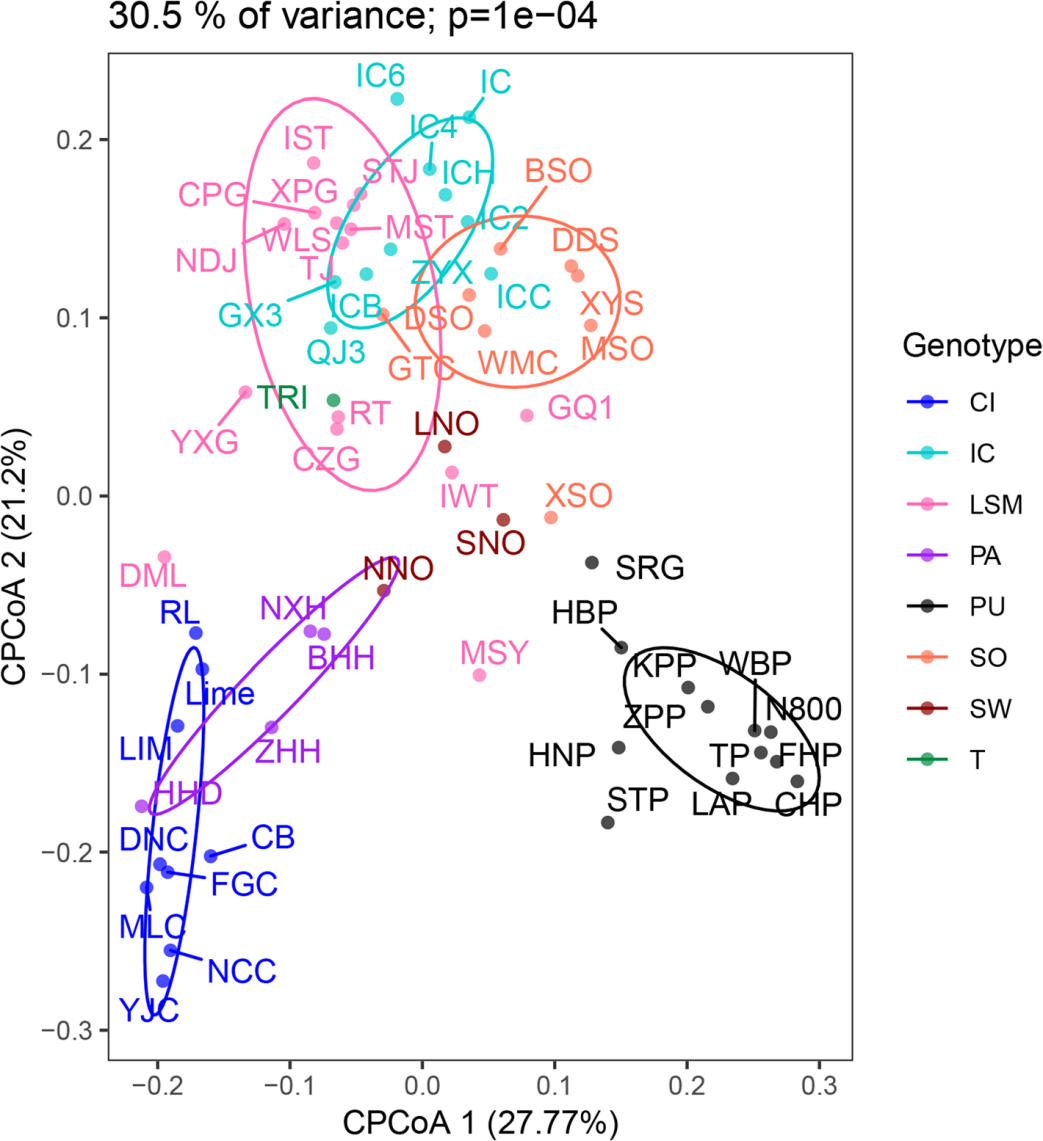
CPCoA of citrus germplasms based on volatile profiles of leaves. LSM: loose-skin mandarin; PA: papeda; CI: citron; SO: sour orange; IC: *C. ichangensis*; SW: sweet orange; PU: pummelo; TRI: *Poncirus trifoliata*

CPCoA grouped the 62 citrus germplasms into six clusters based on the leaf volatiles: cluster 1, loose-skin mandarin; cluster 2, *C. ichangensis*; cluster 3, sour orange; cluster 4, pummelo; cluster 5, papeda; and cluster 6, citron. Generally, different accessions of the same species were clustered together. Three *C. junos* germplasms (GuanXian xiangcheng No.3, Qianjiang xiangcheng No.3 and Ziyang xiangcheng) were clustered with loose-skin mandarin (Fig.2).

### Mono- and Sesquiterpenoid biosynthesis in wild and cultivar Citrus Germplasms

**Fig. 3:**
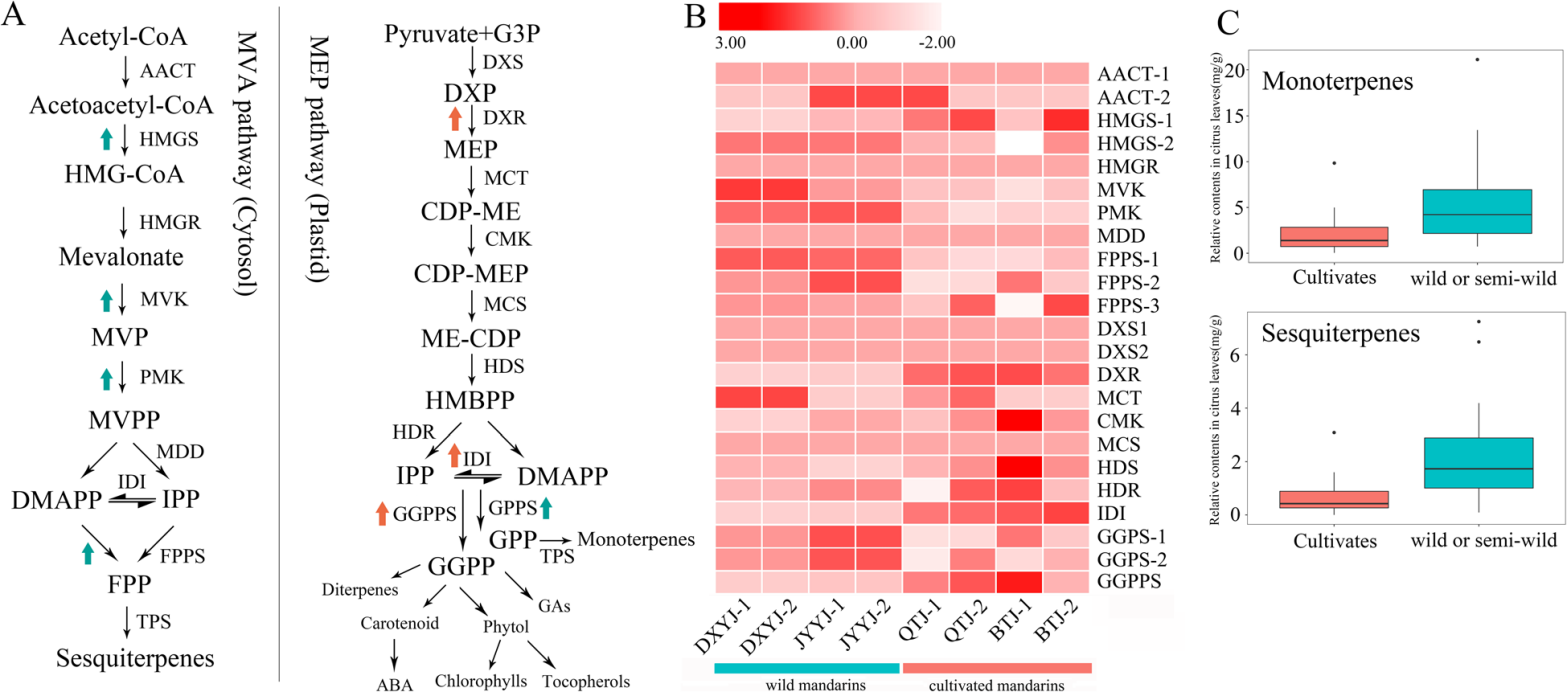
Differential gene expression patterns of two terpenoid biosynthetic pathways in leaves of cultivated citrus species and wild or semiwild germplasms. **a**: biosynthetic pathway of terpenoids in plants. AACT: acetoacetyl-CoA thiolase; HMGS: 3-hydroxy-3-methylglutaryl-CoA synthase; HMGR: HMG-CoA reductase; MVK: mevalonate kinase; PMK: phosphomevalonate kinase; IDI: isopentenyl diphosphate isomerase; DXS:1-deoxy-d-xylulose 5-phosphate synthase; DXR: 1-deoxy-d-xylulose 5-phosphate reductoisomerase; IPP: isopentenyl diphosphate; FPPS: farnesyl pyrophosphate synthase; GPPS: geranyl pyrophosphate synthase; GGPPS: geranylgeranyl diphosphate synthase. **b**: differentially expressed genes in MVA and MEP pathways; **c**: differences in accumulation of monoterpenes and sesquiterpenes in the leaves of wild or semiwild species and cultivars

There were higher levels and a greater variety of volatile compounds, especially monoterpenes and sesquiterpenes, in the leaves of most wild or semiwild germplasms than in the leaves of cultivars (Fig.3c), which was consistent with the volatile profiles of the flavedo and juice sacs (Additional file 6: Figure S3). Comparison of the expression levels of terpenoid-related genes was conducted in two wild germplasms (JYYJ and DXYJ) and two cultivars (BTJ and QTJ). In the wild germplasms, the expression of *3-hydroxy-3-methylglutaryl-CoAsynthase-2* (*HMGS-2*), *mevalonate kinase* (*MVK*), *phosphomevalonate kinase* (*PMK*) and *farnesyl pyrophosphate synthase* (*FPPS*) in the MVA pathway and *geranyl pyrophosphate synthase* (*GPPS*) in the MEP pathway was significantly higher than that in the cultivars. In addition, *geranylgeranyl diphosphate synthase* (*GGPPS*) in the MEP pathway, which is involved in the metabolism of carotenoids, abscisic acid (ABA) and diterpenes, showed significantly lower expression in the wild germplasms than in the cultivars (Fig.3)ab.

**Fig. 4:**
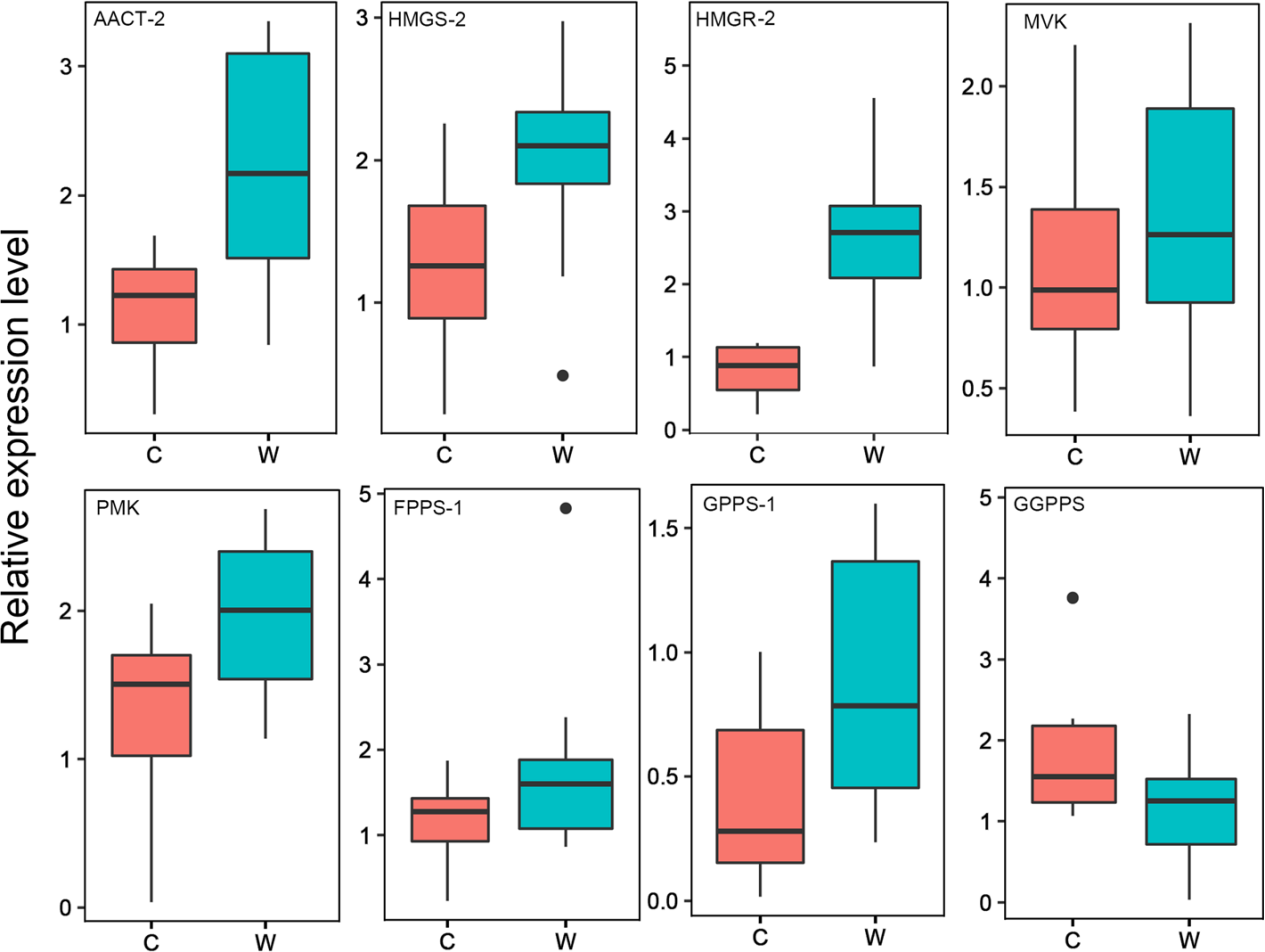
RT-qPCR analysis of the transcript levels of four genes in the MVA pathway and two genes in the MEP pathway in cultivar and wild or semiwild citrus germplasms. Transcript levels are expressed relative to the expression level of the gene encoding the *Actin* extension protein. C: cultivar citrus germplasms; W: wild or semiwild citrus germplasms

To further clarify the expression level of terpenoid-related genes in the MVA and MEP pathways in wild and cultivar germplasms, leaf samples from 10 wild and 10 cultivar citrus germplasms were used for RT-qPCR analysis. (Additional file 7: Table S4). The expression levels of most genes showed the same tendency between the wild and cultivar citrus germplasms by RT-qPCR and transcriptome data analysis. RT-qPCR results showed that the expression levels of most genes in the MVA pathway in the wild citrus germplasms were higher than those in the cultivars, while most genes in the MEP pathway were not significantly different between the wild and cultivar germplasms. In the wild germplasms, the expression of *GPPS* (*GPPS-1 and GPPS-2*) was higher than that in the cultivars, while *GGPPS* showed lower expression in the wild germplasms than in the cultivars (Fig.4 and Additional file 8: Figure S4).

### Volatile profiles in flowers

A total of 82 volatile compounds were detected in citrus flowers from 25 germplasms at two opening stages (balloon stage, F1; fully open stage, F2), including 60 terpenoids and 22 other compounds (Additional file 9: Table S5). The TVC ranged from 1414.33 ± 51.39 μg/g (GQ1, F1) to 6235.60 ± 142.94 μg/g (Star Ruby grapefruit, SRG, F1), and the number of compounds ranged from 19 (Fenghuang pummelo, FHP, F1&F2) to 45 (SRG, F2) (Additional file 9: Table S5). Terpenoids were the most abundant volatiles in citrus flowers, accounting for more than 80% of the TVC in most citrus germplasms, with the exception of red-tangerine (approximately 39%) (Additional file 10: Table S6). The dominant compounds in flowers varied among different species: sabinene was the major compound in sweet orange, followed by *D*-limonene and linalool; γ-terpinene was the most abundant in most loose-skin mandarin germplasms, with the exception of red-tangerine (dimethyl anthranilate); *D*-limonene and *β*-ocimene were dominant in pummelo; and *D*-limonene, *β*-pinene and *β*-ocimene were the most abundant in lemon (Additional file 9: Table S5 and Additional file 10: Table S6). In contrast to the volatiles in leaves, 8-hydroxylinalool was unique to flowers, and the levels of α-sinensal, sabinene, nerolidol and farnesol in flowers were much higher than those in leaves, while the linalool content was similar to that in leaves. However, for most volatiles, such as caryophyllene and germacrene D, lower levels were detected in flowers (Additional file 11: Figure S5).

Within the same germplasm, flower volatile profiles were generally similar between the two investigated stages (Additional file 12: Table S7). However, at the fully open stage, the levels of linalool, 8-hydroxylinalool, nerolidol, and farnesol significantly increased in sweet orange, whereas that of sabinene decreased (Additional file 13: Figure S6). Pummelo showed significant increases in linalool, nerolidol, and farnesol levels, whereas decreases in *β*-myrcene, *β*-pinene and sabinene levels were observed. Loose-skin mandarin and lemon showed increases in farnesol levels (Additional file 9: Table S5).

### DEG profile in Cara Cara navel Orange flowers and characterization of *STPS*

A total of 36 volatile compounds were detected in the flowers of Cara Cara navel orange, including 27 terpenoids (Additional file 9: Table S5), with sabinene as the most abundant compound. The flowers at two opening stages were used for transcriptomic analysis, and a total of 35.8 G high-quality base pairs (at least 5.2 G for each sample) were obtained. Approximately 96.16–98.19% of the total reads were aligned to the sweet orange genome. Among the aligned reads, 89.67–92.41% were uniquely aligned, and 5.64–6.59% were mapped to multiple loci (Additional file 14: Table S8). In total, the expression patterns of 18,654 and 18,267 expressed genes in F1 and F2, respectively, had FPKM values higher than 0.5. The average FPKM values were 33.17 and 44.53, and the expressed genes with FPKM values ranging from 1 to 100 were 86.71 and 85.24% of all the unigenes in F1 and F2, respectively. There were 1013 and 626 genes expressed in only F1 and F2, respectively (Additional file 15: Figure S7).

A total of 2528 differentially expressed genes (DEGs) were identified by RNA-Seq analysis (| Log_2_FC | > 1.5 and *P*-value < 0.05) (Additional file 16: Table S9). GO annotation and enrichment results were mainly associated with plant metabolites, such as lignin, phenylpropanoids, second metabolites and aromatics (Additional file 17: Figure S8). Compared with balloon-stage flowers, 1281 and 1247 genes were significantly up- and downregulated, respectively, in fully open flowers. Among these DEGs, 47 *TPS* and 65 *CYP450* genes were found (Additional file 18: Table S10). Correlation analysis between the terpene profiles and gene expression levels revealed that the content of sabinene and the expression level of *Cs3g04360* had the highest Pearson correlation coefficient (0.95). The Pearson correlation coefficient was higher than 0.9 between linalool content and 10 *TPS* genes (Cs5g23540, Cs5g22980, Cs2g03570, Cs3g21560, Cs2g22180, Cs2g07250, Cs2g07240, Cs2g06470, Cs2g07230 and Cs7g17670) (Additional file 17: Figure S8 and Additional file 18: Table S10). The Pearson correlation coefficient was higher than 0.9 between 8-hydroxylinalool and 36 *CYP450* genes, including seven CYP76C subfamily genes. Those candidate genes maybe contribute to the production of the corresponding volatile terpenes.

According to the high Pearson correlation coefficient, a *TPS* candidate gene named *STPS* in this study was cloned from Cara Cara navel orange flower, with an open reading frame of 1824 bp and encoding a protein of 607 amino acids (Additional file 19: Table S11), which was similar to that encoded by *Cs3g04360* but with 10 different residues (Additional file 20: Figure S9 A). The protein contained the expected divalent-metal-binding region (DDXXD) necessary for TPS activity and the RRX_8_W motif common to cyclic-monoterpene-producing enzymes (Additional file 21: Table S12). The expression of *STPS* significantly decreased in fully open flowers (Additional file 20: Figure S9 B).

**Fig. 5:**
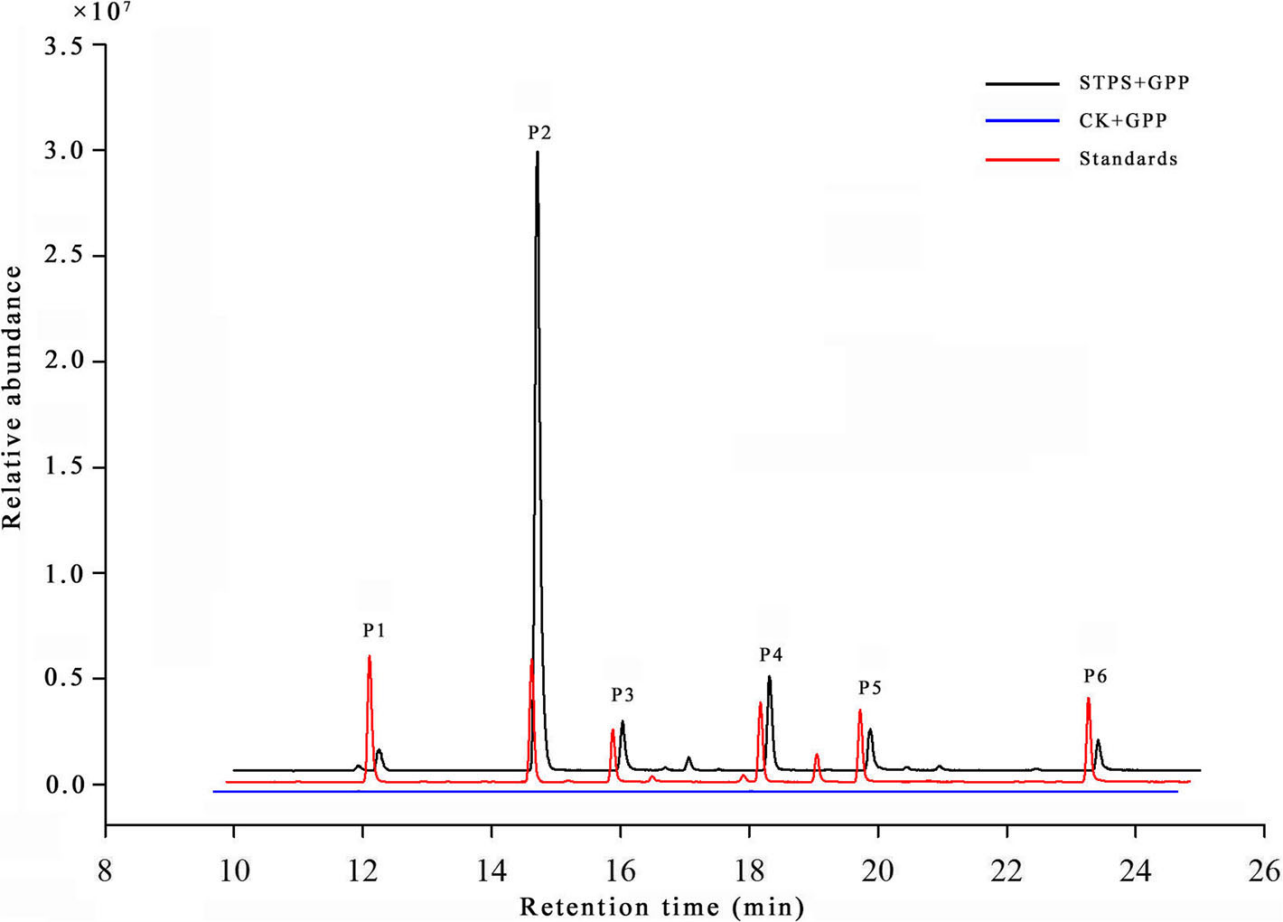
Monoterpene compounds produced by recombinant STPS in vitro. pET-28a(+) + GPP: pET-28a(+) empty vector added with GPP; STPS + GPP: STPS protein added with GPP. Standards: six authentic standards were used for compound identification. P1: α-pinene, P2: sabinene, P3: *β*-myrcene, P4: *D*-limonene, P5: *trans-β*-ocimene, P6: linalool

Recombinant proteins were expressed in *E. coli* and purified using a combination of Ni^2+^ affinity and size exclusion chromatography. Western blot analysis showed that a prokaryotic expression vector that produced a large amount of protein was successfully constructed (Additional file 17: Figure S8 CD). With the addition of GPP or FPP in vitro, GC-MS detection after solvent extraction showed that STPS catalyzed the conversion of GPP to monoterpenes, including 61.26% sabinene, 18.68% *D*-limonene, 7.89% linalool, 5.65% *trans-β*-ocimene, 4.32% *β*-myrcene, and 2.20% α-pinene (Fig. 5). No terpene compounds were detected when FPP was used as a substrate.

## Discussion

### Diverse leaf volatiles in leaves can facilitate studies of Citrus taxonomy

Previous research has indicated that citrus plants, particularly the peels, are rich in volatiles [4, 22]. The TVC was above 10 mg/g in the peels of citrus plants but mostly below 0.05 mg/g in the juice sacs, 3–10 mg/g in the leaves, and 2.5–5 mg/g in the flowers (Additional file 22: Figure S10). Our previous work reported that the number of volatile compounds was 40–60 in the peels and below 20 in the juice sacs [22]. However, the present study identified 30–50 volatile compounds in the leaves and 30–40 volatile compounds in the flowers (Table 1; Additional file 22: Figure S10 and Additional file 9: Table S5). Although the total levels and numbers of compounds detected in leaves and flowers were less than those the peels, some compounds were found to be specific to leaves and flowers, and the dominant compounds in leaves were more diverse than those in the peels.

In most citrus germplasms, monoterpenes were the dominant volatile compounds in the flavedo and juice sacs of fruits, especially *D*-limonene (mostly above 60%) [22, 24]. However, consistent with previous studies [31], *D*-limonene was not the most abundant volatile in the leaves of most citrus germplasms. Notably, linalyl acetate, linalool and *β*-elemene were predominantly accumulated in sour orange and YXG (Additional file 3: Table S3), which were reported to have anti-inflammation and antitumor bioactivities [15, 17].

Additionally, our previous work has revealed that the peel of *C. ichangensis* is rich in sesquiterpenoids. Accordingly, 76 sesquiterpenoids were detected in the leaves of the 10 *C. ichangensis* germplasms, among which seven were specific to *C. ichangensis* (Additional file 1: Table S1). Volatile profiles in leaves might be used for studies of citrus taxonomy. The result of the CPCoA classification based on volatile compounds in citrus leaves was similar to the HCA result based on citrus peel volatiles [22]. Previous research has indicated that sour orange is a hybrid of loose-skin mandarin and pummelo [32]. In this study, sour orange was clustered near loose-skin mandarin, implying that loose-skin mandarin has a strong influence on volatile metabolism in sour orange, which is also consistent with the clustering results for the peel [22]. *C. junos* is a hybrid of *C. ichangensis* and loose-skin mandarin [33]. CPCoA analysis revealed that *C. junos* was clustered between *C. ichangensis* and loose-skin mandarin (Fig.2). The taxonomy of *Citrus mangshanensis* has been disputed in previous reports [28, 32]. This species was clustered adjacent to pummelo in this study (Fig.2), which is similar to the report of Liu et al. [28]. It has been demonstrated that volatiles could be applied in the chemotaxonomic study of citrus plants [34], the reliability of which has been recently verified by additional researchers [22, 27, 28]. Our CPCoA analysis provided further evidence to support the reliability.

In the essential-oil industry, sabinene, which is abundant in sweet-orange leaves and flowers, is the major constituent of carrot seed oil. Linalool, which is abundant in the leaves of most loose-skin mandarin germplasms, has multiple commercial applications, the majority of which are based on its pleasant scent. Linalyl acetate is rich in the leaves of most sour-orange germplasms, and *C. ichangensis* is one of the principal components of the essential oil of bergamot. *D*-limonene, a flavoring agent in food manufacturing, is the most important compound in most citrus peels and is also abundant in citron leaves (Additional file 3: Table S3). Many flavor compounds have been found in citrus leaves, some of which can be used in food additives, cosmetics and perfumes.

Furthermore, as leaves are the main photosynthetic organs, leaf volatiles might play a role in repelling pests and harmful microorganisms [11, 12]. Considering the fast growth, high biomass and four-season harvesting time span of citrus leaves, the composition and biosynthetic mechanisms of volatiles in citrus leaves warrant further in-depth research for industrial applications.

### Decreased abundance of leaf volatiles in Citrus cultivars is due to a negative effect of Citrus domestication

The total levels of monoterpenes and sesquiterpenes in wild or semiwild germplasms in various tissues were higher than those in cultivars, especially in leaves. Hence, the levels of monoterpenes and sesquiterpenes have possibly decreased during domestication. Transcriptomic and RT-qPCR analyses of the expression levels of key genes in wild and cultivar tangerine leaves revealed that most of the genes (*HMGS-2*, *MVK*, *PMK* and *FPPS*) in the MVA pathway and *GPPS* in the MEP pathway had significantly higher expression levels in wild tangerine than in cultivars. However, *GGPPS* in the MEP pathway had a lower expression level in wild tangerine than in cultivars (Fig.3; Fig.4). The different expression levels of these genes in the MVA and MEP pathways may affect the accumulation of monoterpenes and sesquiterpenes in wild and cultivar tangerines. It has been reported that *GGPPS* is responsible for much of the metabolism of some compounds, such as the maturation-related ABA and color-related carotenoids [35]. During the long-term process of citrus breeding/selection, great importance has been attached to fruit size, color, yield, sugar-acid ratio and maturation season. As a result, the organic acid levels of citrus were significantly reduced during the process of artificial selection and domestication [36]. The color of fruits has always been a target of artificial breeding, and increased levels of carotenoids might have resulted in decreased levels of volatile compounds in citrus plants.

### Candidate genes found in Citrus flowers account for their distinct aromas

In comparison with volatiles in citrus peels and leaves, those in the entomophilous flowers of citrus plants were lower in abundance and fewer in number. The increased levels of linalool, 8-hydroxylinalool, nerolidol, and farnesol and decreased levels of some monoterpenes (Additional file 13: Figure S6) at the fully open flower stage are closely related to the sweet floral aroma (http://www.thegoodscentscomp-any.com/index.html) and the attraction of specific insects for pollination, which are of vital importance in both theoretical and practical research.

There were 95 candidate *TPS* loci found in sweet orange, but very few of these loci have been verified [18, 19, 21]. Some terpenoids were significantly different at the two opening stages of flowers, with a high Pearson correlation coefficient observed with some *TPS*s and *CYP450*s by transcriptomic and correlation coefficient analysis, such as two *TPS* genes (Cs3g04360 and orange1.1 t00017), which may function in the production of sabinene (Additional file 17: Figure S8). Functional analysis of STPS in vitro revealed that this enzyme is a monoterpene synthase that produces six monoterpenes, among which sabinene was the main product when GPP was the substrate (Fig.5), indicating that *STPS* is an important gene in the synthesis of sabinene.

## Conclusions

The volatile compounds from citrus leaves and flowers were identified in this study. Our results suggested that 31 important terpenoids were abundant in wild or semiwild germplasms. Transcriptomic and RT-qPCR analyses revealed that the expression levels of most genes in wild or semiwild germplasms were higher than those in cultivars, possibly because of a negative effect of domestication on the volatile terpene biosynthase in citrus leaves. The sweet smell of fully open flowers may be attributed to increased levels of four terpene alcohols. Our findings indicated that citrus leaves can be a valuable raw material for essential-oil production and studies of the biosynthesis of terpenoids and regulation of terpenoid metabolism.

## Methods

### Materials and sample collection

Leaves of 62 citrus germplasms were collected from four citrus production areas in China in 2016,including 16 from the National Citrus Breeding Center (NCBC) (Wuhan, Hubei), 40 from the Citrus Research Institute, Chinese Academy of Agricultural Sciences (Beibei, Chongqing), three from the citrus research institute of Ruili (Ruili, Yunnan) and three from the Guangdong Academy of Agricultural Sciences (Guangzhou, Guangdong). There were 15 Loose-skin mandarins (*Citrus reticulata*), three Sweet oranges (*C. sinensis*), 12 Pummelos (*C. maxim*), 10 *C. ichangensis*, nine Citrons (*C. lemon*), four Papedas (*C. hystrix*), eight Sour oranges (*C. aurantium*) and one *Poncirus trifoliata* (Table 1). The fully expanded leaves on new spring shoots were collected in orchards under normal management in July. A total of 30–40 average-sized adult leaves per germplasm were washed with tap water, wiped dry with clean paper and then randomly divided into three biological replicates. The leaves were placed in liquid nitrogen and stored at − 80 °C for further analysis.

Flowers of 25 germplasms (Additional file 9: Table S5) at two opening stages (the 4th day of balloon stage and the 2nd day of the fully open stage) were collected from the outer part of the canopy of adult healthy trees in the NCBC in 2017. A total of 12–15 flowers were randomly divided into three replicates per germplasm, placed in liquid nitrogen and then stored at − 80 °C for further analysis.

### Standards and reagents

For the determination of volatile compounds on GC-MS, 57 authentic standards were purchased from Sigma (St.Louis, MO, USA) and Alfa (Alfa Aesar Co. Ltd. UK) as shown in Additional file 2: Table S2, which were dissolved in Methyl tert-butyl ether (MTBE, HPLC grade). MTBE was purchased from Tedia (Fairfield, OH, USA) and used as solvent to extract the volatiles.

### Volatile extraction and GC-MS analysis

Volatile extraction was performed according to Zhang et al. [22] with minor modifications. The leaves or whole flowers were ground into powder. Then, 0.3 g of sample was used for the extraction of volatiles. The profiles of volatiles were analyzed by TRACE GC Ultra GC coupled with a DSQ 8000 mass spectrometer (Thermo Fisher Scientific, Waltham, MA) with a TRACE TR-5 MS column (30 m × 0.25 mm × 0.25 μm; Thermo Scientific, Bellefonte, PA) with a split ratio of 20:1. Other parameters for GC-MS of volatiles were based on the study of Liu et al. [24].

### RNA extraction and Transcriptomic sequencing

Following the method of Cao et al. [37], total RNA was extracted from the flowers of Cara Cara navel orange at the balloon (F1–1, F1–2 and F1–3) and fully open (F2–1, F2–2 and F2–3) stages with three biological replicates. For each sample, 2 μg of total RNA was sent to Millennium Co. (Seoul, Korea) for RNA-Seq library construction and sequencing. The mRNA was purified from the total RNA using the Illumina TruSeq RNA Sample Prep Kit v2 and assessed using an Agilent Technologies 2100 Bioanalyzer (Agilent, United States). Following the TruSeq RNA Sample Preparation v2 Guide (15026495) (Illumina, United States), first- and second-strand complementary DNA were synthesized, and then, double-strand cDNA was purified, and adapters were added. The mRNA was cleaved into short fragments of approximately 300 bp, and the constructed RNA libraries were sequenced on the Illumina HiSeq 4000 platform in paired-end mode.

### RNA-Seq data analysis

The RNA-Seq data of F1 and F2 were newly sequenced in this study. RNA-Seq raw data for the leaves of two wild citrus germplasms (DXYJ, SRR5807703; JYYJ, SRR5807742) and two cultivars (BTJ, SRR5807774; QTJ, SRR5807788) [36] were downloaded from NCBI (https://www.ncbi.nlm.nih.gov/). The reference citrus genome V2.1 and annotation files were downloaded from the *Citrus sinensis* annotation project (http://citrus.hzau.edu.cn/orange/index.php). The index of the reference genome was generated by STAR_2.6.0a [38]. All RNA-Seq rawdata were filtered to remove the low-quality reads using Trimmomatic-0.36 [39]. Cleaned reads were mapped to the reference citrus genome using STAR_2.6.0a [38]. Based on the alignments, transcript abundance was estimated using HTSeq [40]. Analysis of DEGs was performed using the R package edgeR [41]. F1 and F2 were used to identify the candidate genes for terpene synthase. DXYJ, JYYJ, BTJ and QTJ were used to analyze the expression patterns of genes in the MVA and MEP pathways.

### RNA extraction and RT-qPCR

Total RNA was extracted from 20 citrus leaf samples (Additional file 7: Table S4) using the EASYspin Plus Plant RNA Kit (RN38, Aidlab). First-strand cDNA was synthesized using the HiScript® II Q RT SuperMix for qPCR (+gDNA Wiper, Vazyme). The gene-specific primers used for RT-qPCR are listed in Additional file 21: Table S12. RT-qPCR was performed using the Roche LightCycler 480 system with 384-well plates using the Hieff™ qPCR SYBR Green Master Mix (No Rox, Yeasen Biotech Co., Ltd., Shanghai, China), and the program was performed according to the manufacturer’s protocol. *Actin* was used as the endogenous control. RT-qPCR data analysis was performed as described by Lu et al. [42].

### *STPS* gene identification and functional analysis

*STPS* was cloned using the primers and vectors described in Additional file 21: Table S12. The open reading frame and transit peptide were predicted using ORF Finder (http://www.ncbi.nlm.nih.gov) and TargetP (http://www.cbs.dut.dk/service/ TargetP), respectively. Western blot analysis of STPS was conducted as described by Cao et al. [37].

For functional analysis, *STPS* was amplified to remove the chloroplast-targeting peptide and cloned into the pET-28a(+) vector with primers listed in Additional file 21: Table S12. Recombinant N-terminal His-tagged proteins were expressed in *Escherichia coli* BL21(DE3). The bacterial strain was grown to A_600_ = 0.6 at 37 °C in 100 ml of LB medium with 50 μg/ml kanamycin. Cultures were induced with 1 mM isopropyl 1-thio-β-D-galactopyranoside (IPTG) and incubated with shaking for 16 h at 180 rpm and 16 °C. The proteins were purified by Ni^2+^ affinity and size exclusion chromatography as described previously. STPS was characterized in vitro as described by Brillada et al. [43] with minor modifications. STPS was added to 500 μl of assay buffer (25 mM HEPES (pH 7.3), 10 mM MgCl_2_, 0.1 mM MnCl_2_, 0.2 mM NaWO_4_, 0.1 mM NaF, 5 mM DTT, and 10% glycerol), mixed with 50 μM GPP or FPP, covered with pentane and incubated at 30 °C for 30 min. The pentane layer was transferred to a glass vial and subjected to GC-MS analysis as described by Liu et al. [24].

### Data analysis

For identification, 57 standard volatile compounds were used. For those without authentic standards, the identification was based on Xcalibur software and the NIST Mass Spectral Library (NIST 2015). The concentration of each volatile compound was quantified based on comparison with the internal standards. The volatile compound content in citrus peels and juice sacs was obtained from our previous results, as reported by Zhang et al. [22].

Scatter plots were generated using Excel (Microsoft, Seattle, WA). Histograms and boxplots were constructed using the ggplot2 package in R [44] and Sigmaplot 12.0, and the heat map was constructed using TBtools [45]. CPCoA analysis was conducted using the vegan package in R [46]. Differential accumulation of volatile compounds in F1 and F2 of sweet orange was analyzed using the mixOmics (PLS-DA) and ggplot2 packages in R [47]. Correlation coefficient analysis of the terpene profiles and gene expression levels were performed using the PerformanceAnalytics package in R [48].

## Supplementary information

**Additional file 1: Table S1.** Volatiles in citrus leaves (μg/g). U: undetectable.

**Additional file 2: Table S2**. Fifty-seven standards used in volatile compound determination. ^a^Sigma, Sigma-Aldrich (St. Louis, MO); Alfa, Alfa Aesar Co. Ltd. (Heysham, UK).

**Additional file 3: Table S3**. Top 5 high-content volatiles in leaves of citrus germplasms, including *Citrus hystrix*, *C. ichangensis*, citron, pummelo, loose-skin mandarin, sweet orange, sour orange and *Poncirus trifoliata*.

**Additional file 4: Figure S1**. Proportions of monoterpenes and sesquiterpenes in total volatiles in the leaves of various germplasms. LSM: loose-skin mandarin; PA: papeda; CI: citron; SO: sour orange; IC: *C. ichangensis*; SW: sweet orange; PU: pummelo; T: *Poncirus trifoliata*.

**Additional file 5: Figure S2**. Total volatiles in loose-skin mandarin leaves (mg/g).

**Additional file 6: Figure S3**. Total volatile levels and number of compounds in the peels and juice sacs of wild or semiwild species and cultivars (mg/g).

**Additional file 7: Table S4**. Leaves of citrus germplasms were used to RT-qPCR analysis.

**Additional file 8: Figure S4**. RT-qPCR analysis of the transcript levels of eight genes in the MVA pathway and ten genes in the MEP pathway in cultivar and wild or semiwild citrus germplasms. Transcript levels are expressed relative to the expression level of the gene encoding the *Actin* extension protein. C: cultivar citrus germplasms; W: wild or semiwild citrus germplasms.

**Additional file 9: Table S5**. Volatiles in citrus flowers (μg/g). F1: balloon stage, F2: fully open stage. U: undetectable.

**Additional file 10: Table S6**. Summary of important volatiles and terpenoids in flowers. (1): Most abundant volatiles in flowers; (2): proportions of monoterpenes in total volatiles; (3): proportions of sesquiterpenes in total volatiles; (4): proportions of other compounds in total volatiles.

**Additional file 11: Fig. S5**. Levels of volatiles in leaves and flowers (μg/g).

**Additional file 12: Table S7**. Total volatile levels and compounds detected in flowers at two opening stages. F1: balloon stage; F2: fully open stage.

**Additional file 13: Figure S6**. PLS-DA of the volatiles in flowers. A: PLS-DA score plots of flowers. The result of the PLS-DA clearly distinguished the flowers at two opening stages using the volatile profile. B: VIP scores of volatiles in the PLS-DA model for discrimination. C: levels of biomarker compounds in flowers at two opening stages. The levels of the compounds were normalized to Log_2_. F1: balloon stage, F2: fully open stage.

**Additional file 14: Table S8**. Basic statistics of RNA-Seq raw data.

**Additional file 15: Figure S7**. Basic information about the transcriptomic data of flowers. A: expression levels of genes based on RNA-Seq data from the flowers. B: tissue-specific expression of genes in flowers at two opening stages. C: boxplot showing the expression levels of genes from flowers. The raw FPKM data was normalized to Log_10_. F1: balloon stage, F2: fully open stage.

**Additional file 16: Table S9**. Differentially expressed genes between the balloon (F1) and fully open (F2) flower stages.

**Additional file 17: Figure S8**. Differentially expressed genes in flowers at two opening stages. A: GO annotation of the differentially expressed genes. B: correlation coefficient analysis of the terpene content and the expression levels of *TPS* genes. C: relationship between volatile profiles and the expression levels of TPS genes by Cytoscape_3.7.2. The volatile compounds and *TPS* genes are listed in Additional file 18: Table S10.

**Additional file 18: Table S10**. Expression levels of *TPS* genes, *CYP450* genes and the volatile compound content in flowers.

**Additional file 19: Table S11**. cDNA and protein sequences of *STPS*. The cDNA sequence of the chloroplast-targeting peptide is labeled with gray shading; the conserved domain in the protein sequence is labeled with turquoise shading.

**Additional file 20: Figure S9**. A: alignment of deduced amino acid sequences between STPS and four *D*-limonene synthase genes. The amino acid sequences of KU746814, AB110637, AB266584 and Cs3g04360 were obtained from the NCBI website and the genome sequence of Valencia sweet orange. B: sabinene content and relative expression level of *STPS.* C: purification of His-tagged STPS protein analyzed by SDS-PAGE in *E.coli* and Western blot analysis. M: Protein Ladder (#SM0671); 1: STPS-tr flow-through of the lysate from a Ni-NTA His-Bind Resin column; 2, 3: eluate of STPS; 4: STPS-fl flow-through of the lysate from a Ni-NTA His-Bind Resin column; 5, 6: eluate of STPS-fl. D: Identification of recombinant STPS-tr and STPS-fl by Western blot analysis.

**Additional file 21: Table S12**. Primers used in the cloning of *STPS* and construction of expression vectors.

**Additional file 22: Figure S10**. Total volatile content in different citrus tissues (mg/g).

## Data Availability

All data generated or analyzed during this study were included in this published article and the additional files. The RNA-seq data are available from the NCBI under the accession number PRJNA579049.

## Abbreviations

CPCoA: Constrained principal coordinate analysis
CYP450: Cytochrome P450
DEGs: Different expression genes
DMAPP: Isomer dimethylallyl diphosphate
FPP: Farnesyl pyrophosphate
FPPS: FPP synthase
GC-MS: Gas Chromatography–Mass Spectrometry
GGPPS: Geranylgeranyl diphosphate synthase
GPP: Geranyl pyrophosphate
GPPS: GPP synthase
HCA: Hierarchical Cluster Analysis
HMGS: 3-hydroxy-3-methylglutaryl-CoA synthase
IPP: Isopentenyl pyrophosphate;
MEP: 2-C-methyl-D-erythritol-4-phosphate
MTBE: Methyl tert-butyl ether
MVA: Mevalonate
MVK: Mevalonate kinase
PCA: Principal Component Analysis
PLS-DA: Partial Least Squares-Discriminant Analysis
PMK: Phosphomevalonate kinase
STPS: Sabinene synthesis gene
TPS: Terpene synthesis gene
TVC: Total volatile content
