# Supplementary figures and images for: Transcriptomic and metabolomic analyses provide insight into the volatile compounds of citrus leaves and flowers
Source: BMC Plant Biol. 2020 Jan 6;20:7. doi: 10.1186/s12870-019-2222-z (PMC6945444; doi:10.1186/s12870-019-2222-z)

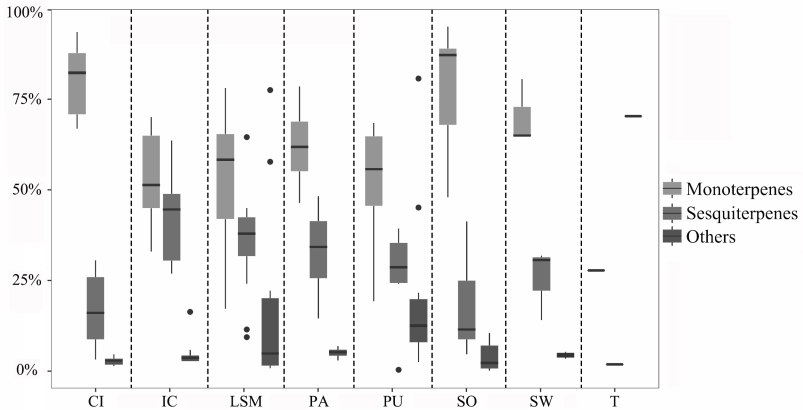

Supplement: Supplementary file 4 — Additional file 4: Figure S1. Proportions of monoterpenes and sesquiterpenes in total volatiles in the leaves of various germplasms. LSM: loose-skin mandarin; PA: papeda; CI: citron; SO: sour orange; IC: C. ichangensis; SW: sweet orange; PU: pummelo; T: Poncirus trifoliata. [file 12870_2019_2222_MOESM4_ESM.pdf]

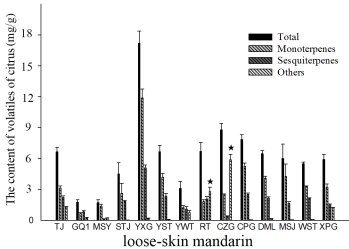

Supplement: Supplementary file 5 — Additional file 5: Figure S2. Total volatiles in loose-skin mandarin leaves (mg/g). [file 12870_2019_2222_MOESM5_ESM.pdf]

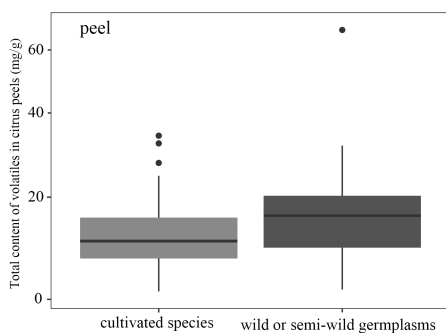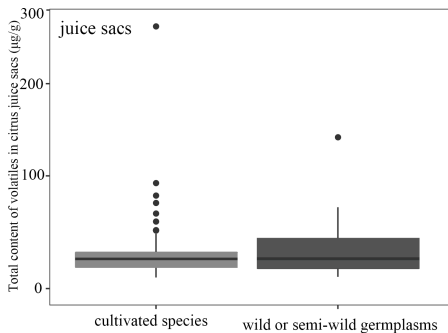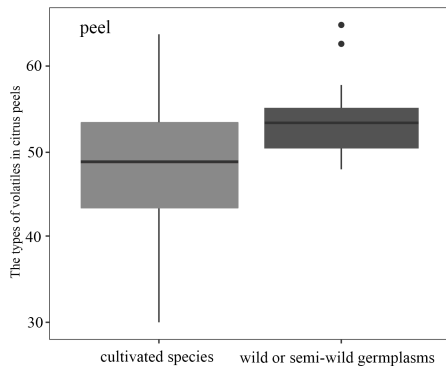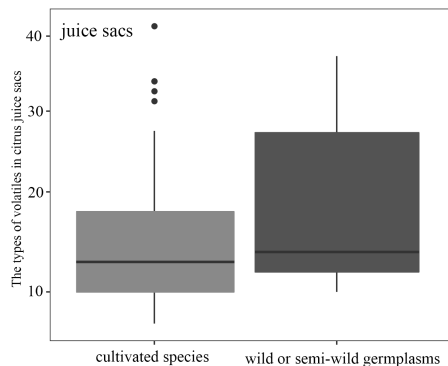

Supplement: Supplementary file 6 — Additional file 6: Figure S3. Total volatile levels and number of compounds in the peels and juice sacs of wild or semiwild species and cultivars (mg/g). [file 12870_2019_2222_MOESM6_ESM.pdf]

Relative expression level

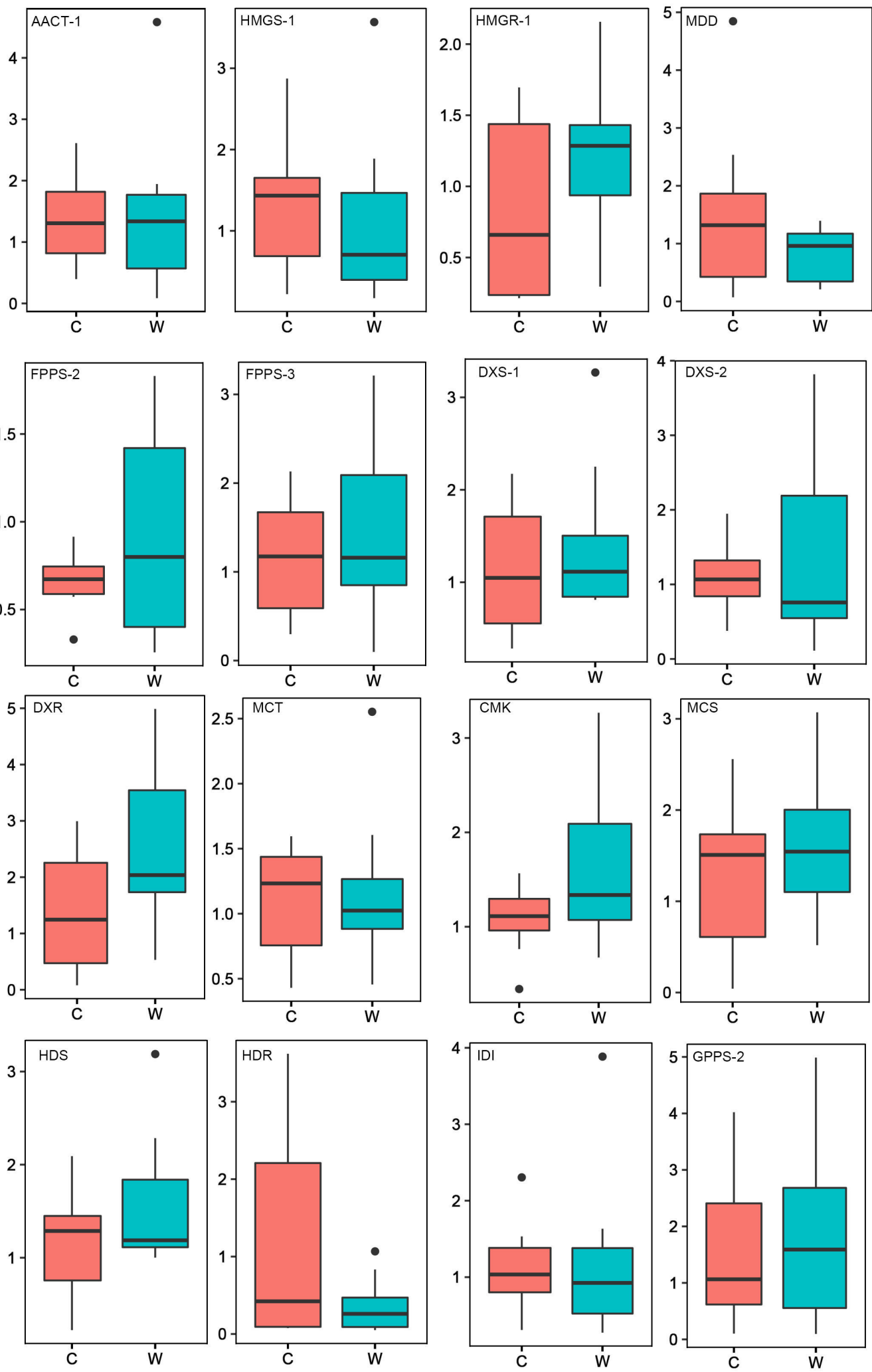

Supplement: Supplementary file 8 — Additional file 8: Figure S4. RT-qPCR analysis of the transcript levels of eight genes in the MVA pathway and ten genes in the MEP pathway in cultivar and wild or semiwild citrus germplasms. Transcript levels are expressed relative to the expression level of the gene encoding the Actin extension protein. C: cultivar citrus germplasms; W: wild or semiwild citrus germplasms. [file 12870_2019_2222_MOESM8_ESM.pdf]

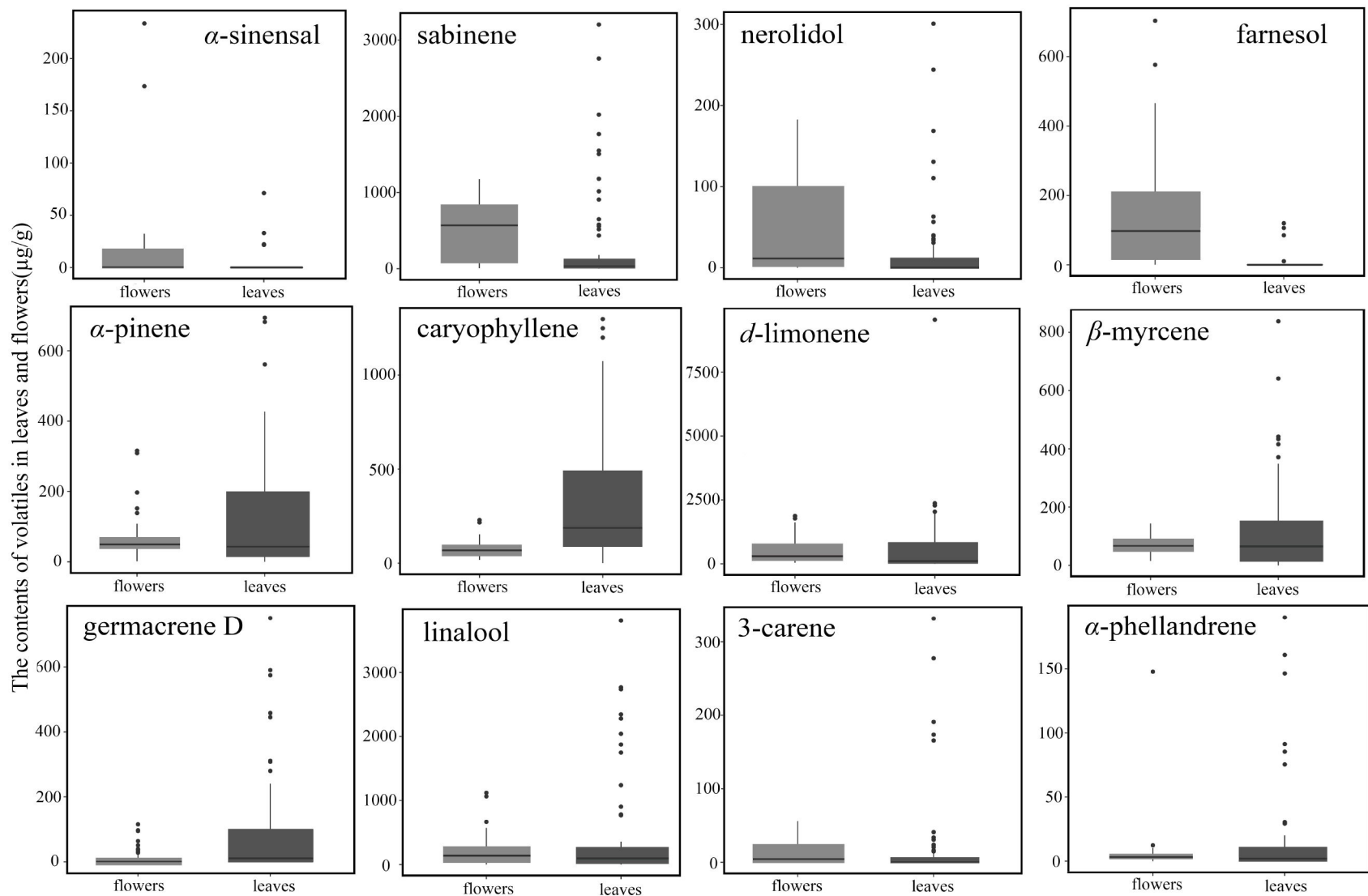

Supplement: Supplementary file 11 — Additional file 11: Fig. S5. Levels of volatiles in leaves and flowers (μg/g). [file 12870_2019_2222_MOESM11_ESM.pdf]

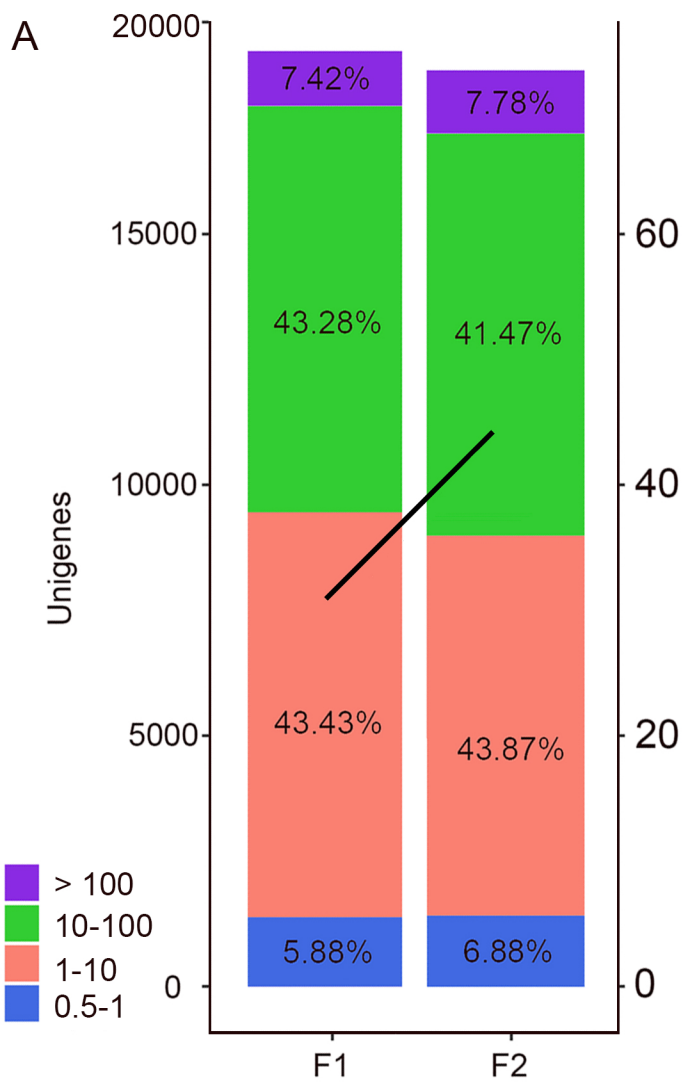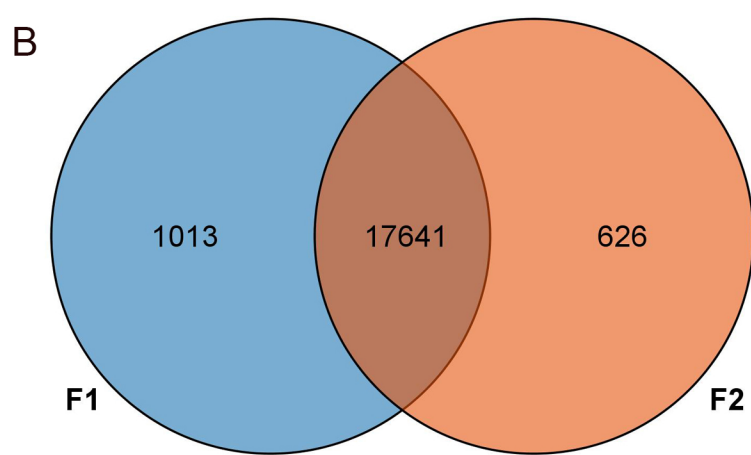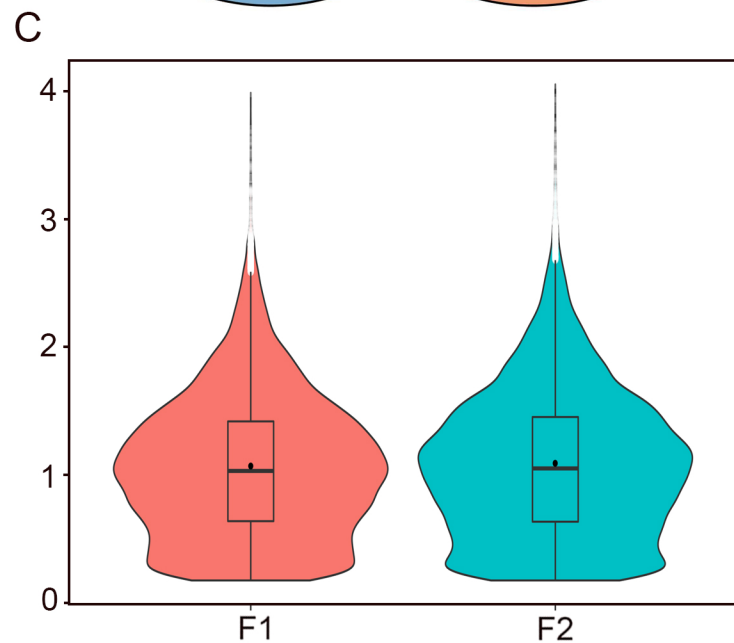

Supplement: Supplementary file 15 — Additional file 15: Figure S7. Basic information about the transcriptomic data of flowers. A: expression levels of genes based on RNA-Seq data from the flowers. B: tissue-specific expression of genes in flowers at two opening stages. C: boxplot showing the expression levels of genes from flowers. The raw FPKM data was normalized to Log10. F1: balloon stage, F2: fully open stage. [file 12870_2019_2222_MOESM15_ESM.pdf]

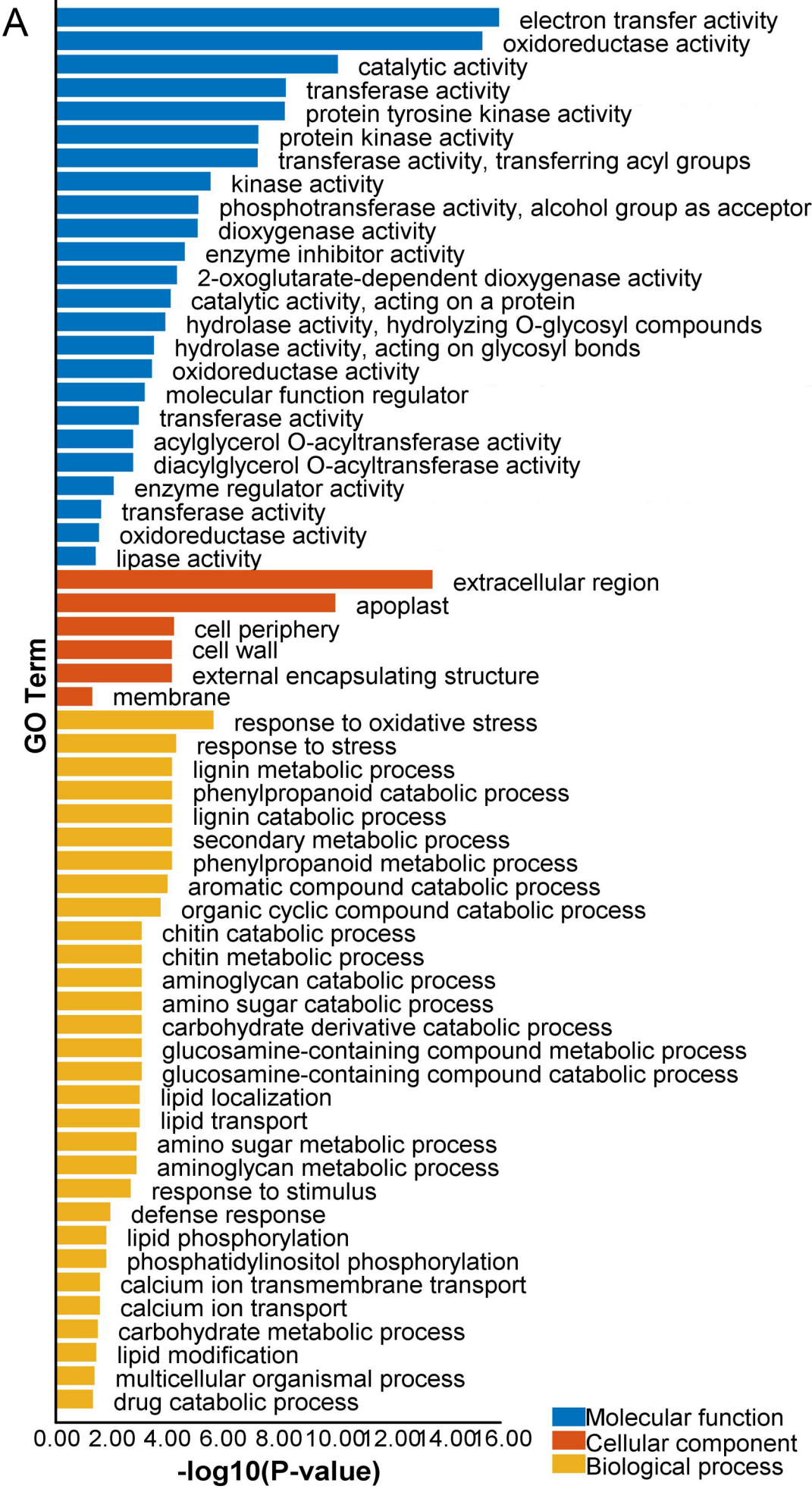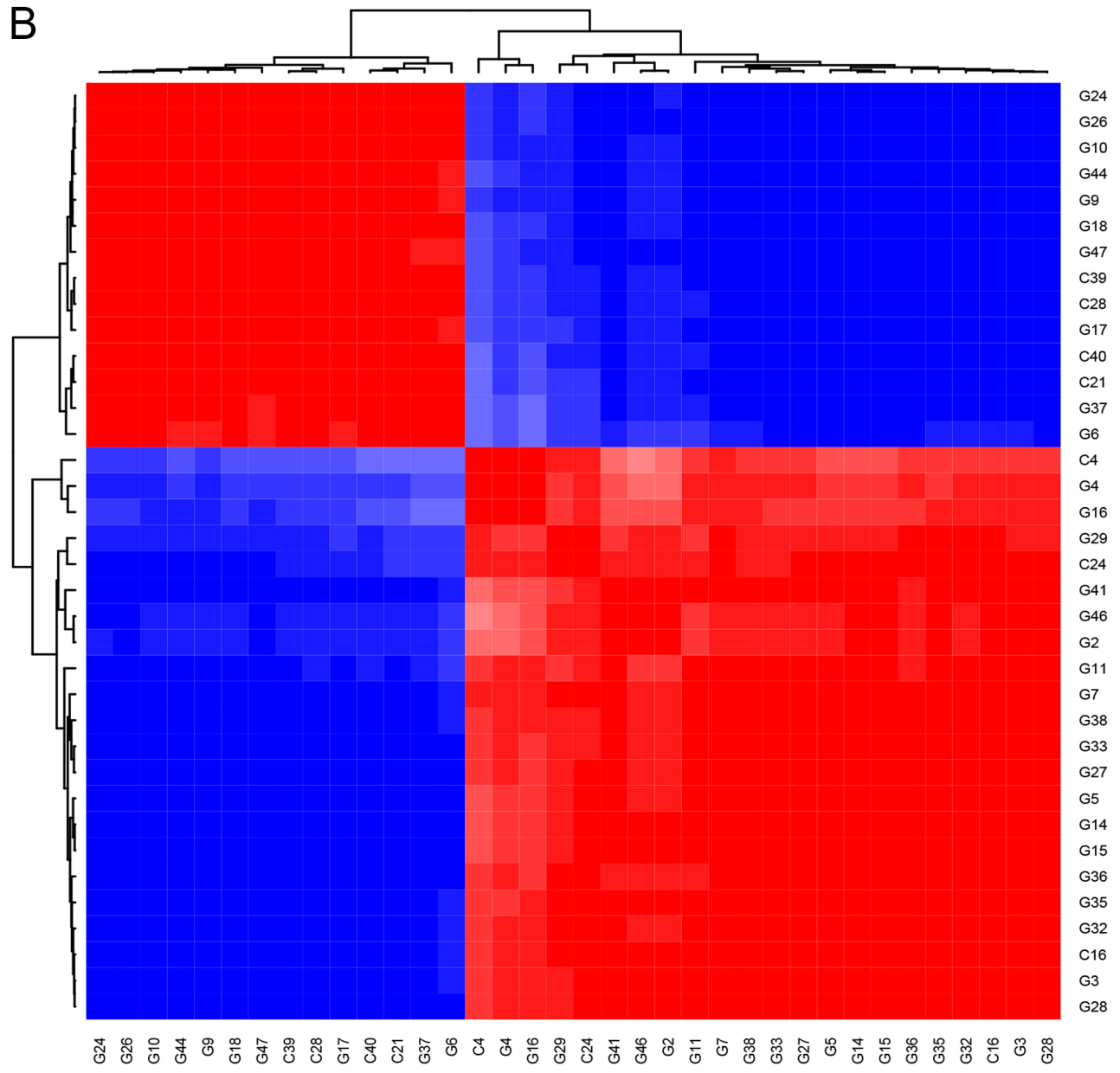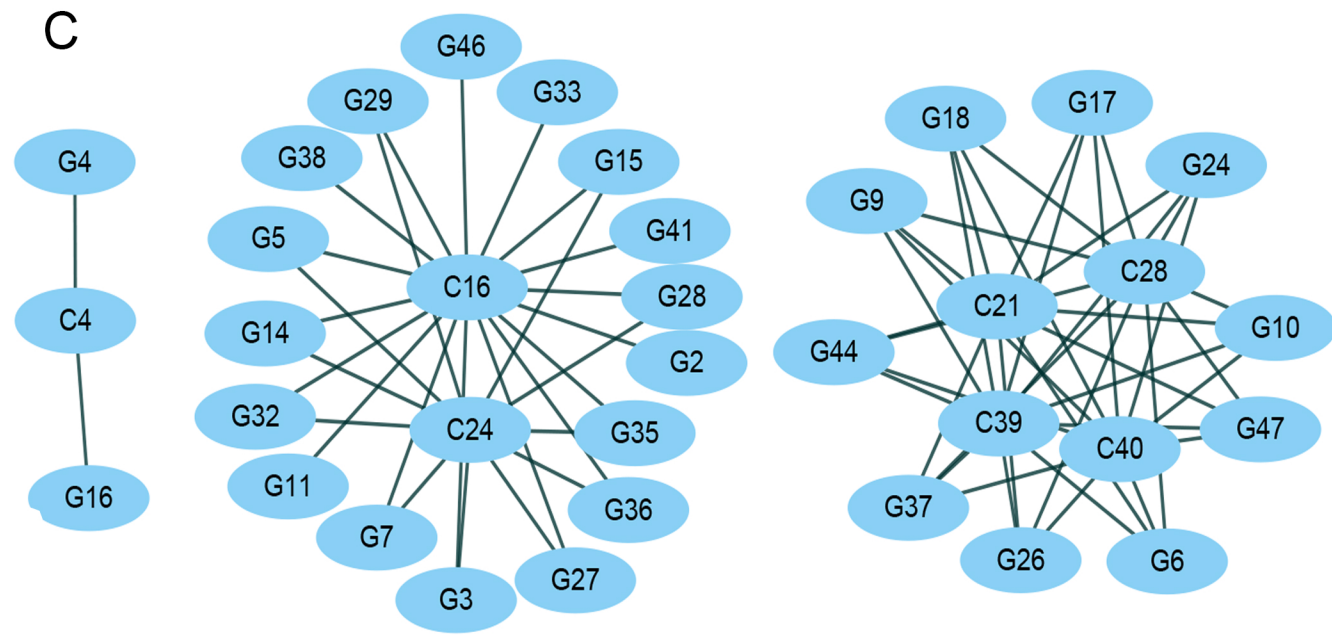

Supplement: Supplementary file 17 — Additional file 17: Figure S8. Differentially expressed genes in flowers at two opening stages. A: GO annotation of the differentially expressed genes. B: correlation coefficient analysis of the terpene content and the expression levels of TPS genes. C: relationship between volatile profiles and the expression levels of TPS genes by Cytoscape_3.7.2. The volatile compounds and TPS genes are listed in Additional file 18: Table S10. [file 12870_2019_2222_MOESM17_ESM.pdf]

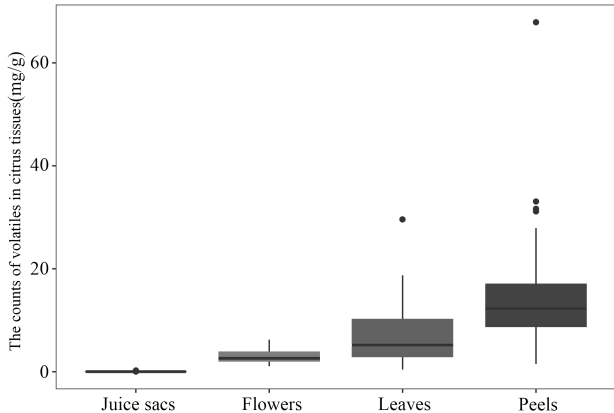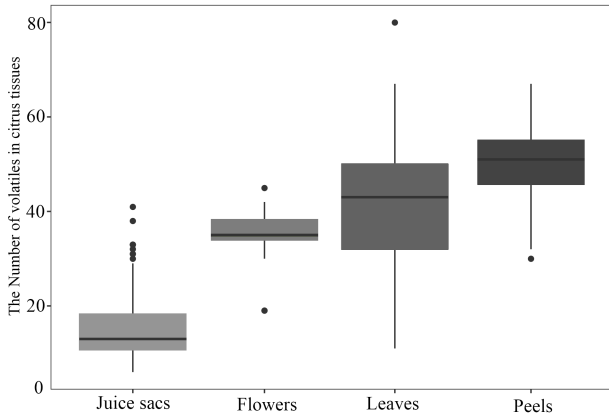

Supplement: Supplementary file 22 — Additional file 22: Figure S10. Total volatile content in different citrus tissues (mg/g). [file 12870_2019_2222_MOESM22_ESM.pdf]
