# Supplementary material for: Transcriptomic and metabolomic analyses provide insight into the volatile compounds of citrus leaves and flowers
Source: BMC Plant Biol. 2020 Jan 6;20:7. doi: 10.1186/s12870-019-2222-z (PMC6945444; doi:10.1186/s12870-019-2222-z)

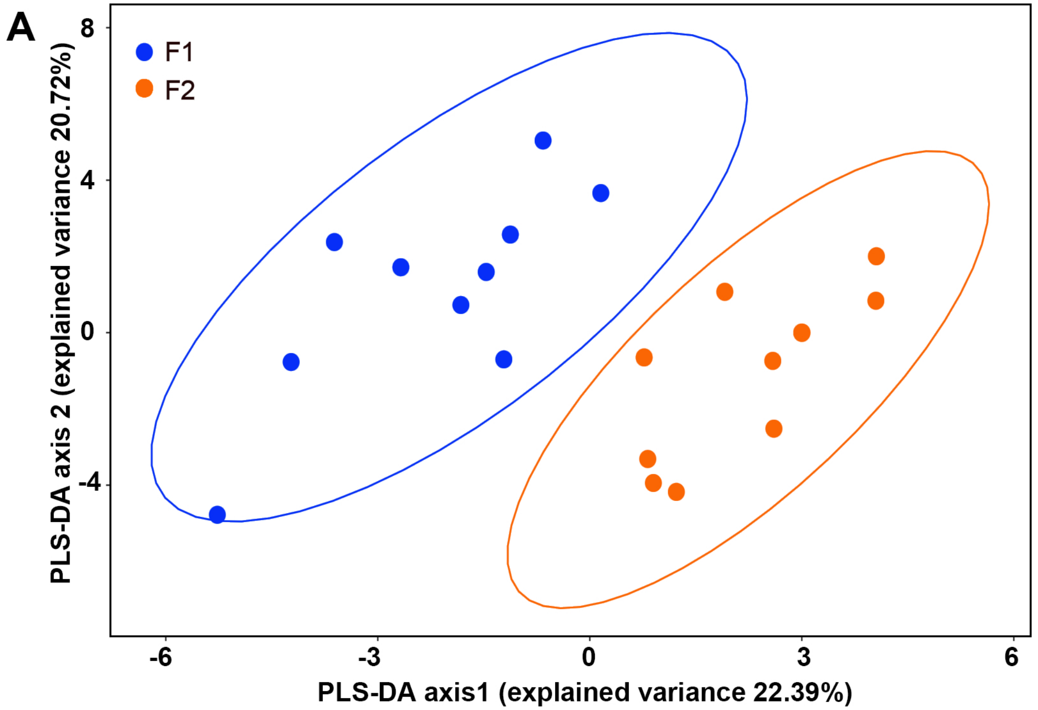

B

Potential chemical markers in flowers

ID	compounds	VIP
C28	8-hydroxylinalool	2.37
C39	nerolidol	2.32
C40	farnesol	1.70
C4	sabinene	1.60
C24	α-terpineol	1.45
C16	*cis*-*β*-terpineol	1.44
C6	*β*-myrcene	1.32
C46	(*Z*)-2-hexen-1-ol	1.18
C21	linalool	1.18
C5	*β*-pinene	1.16
C7	α-phellandrene	1.09
C44	2-hexenal	1.04
C43	(*E*)-2-hexenal	1.00

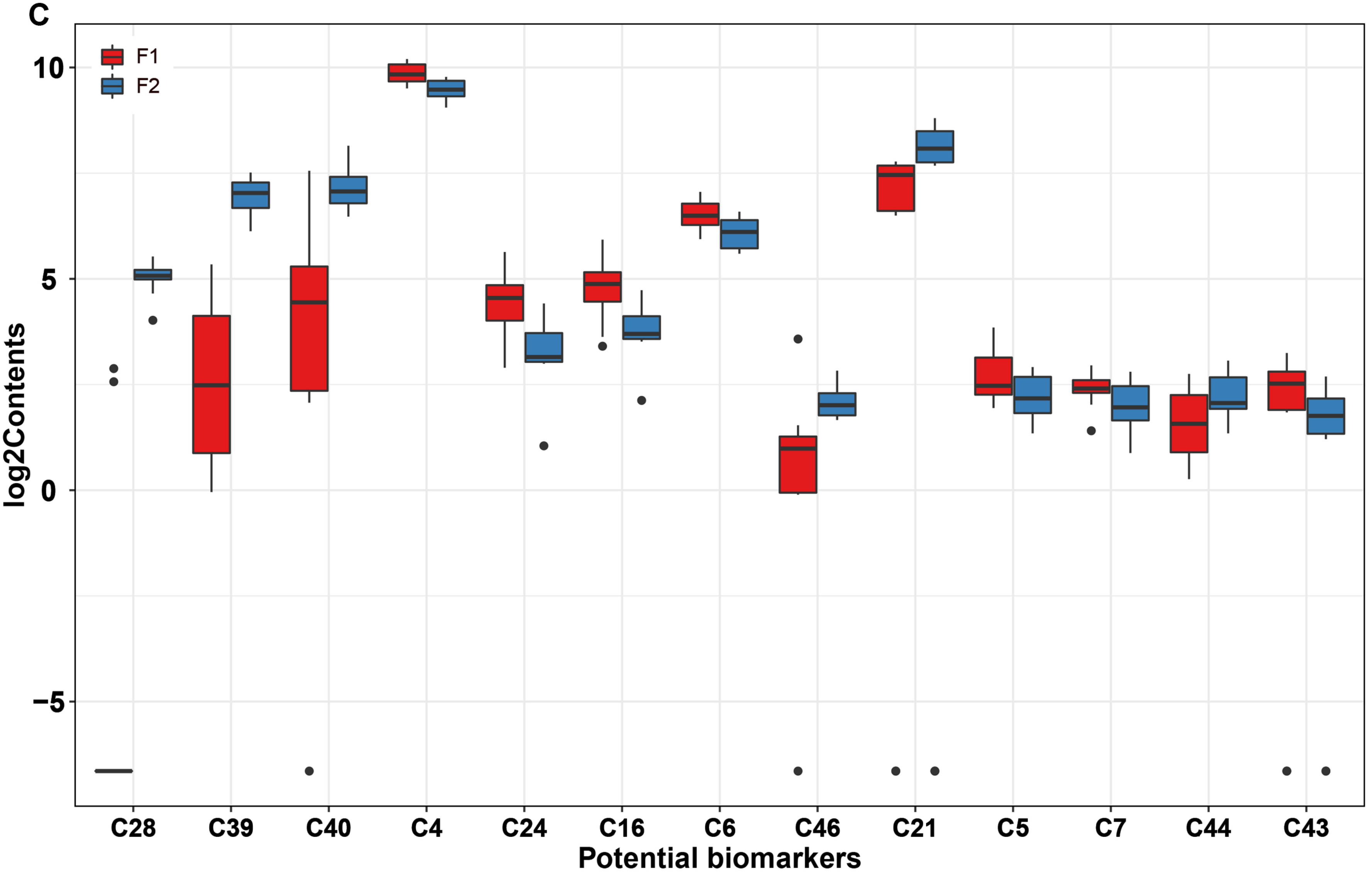

Supplement: Supplementary file 13 — Additional file 13: Figure S6. PLS-DA of the volatiles in flowers. A: PLS-DA score plots of flowers. The result of the PLS-DA clearly distinguished the flowers at two opening stages using the volatile profile. B: VIP scores of volatiles in the PLS-DA model for discrimination. C: levels of biomarker compounds in flowers at two opening stages. The levels of the compounds were normalized to Log2. F1: balloon stage, F2: fully open stage. [file 12870_2019_2222_MOESM13_ESM.pdf]
