# Supplementary material for: Transcriptomic and metabolomic analyses provide insight into the volatile compounds of citrus leaves and flowers
Source: BMC Plant Biol. 2020 Jan 6;20:7. doi: 10.1186/s12870-019-2222-z (PMC6945444; doi:10.1186/s12870-019-2222-z)
Supplement: Supplementary file 19 — Additional file 19: Table S11. cDNA and protein sequences of STPS. The cDNA sequence of the chloroplast-targeting peptide is labeled with gray shading; the conserved domain in the protein sequence is labeled with turquoise shading. [file 12870_2019_2222_MOESM19_ESM.doc]

**Table S11**: cDNA and protein sequences of *STPS*.

> STPS-cDNA

ATGTCTTCTTGCATTAATCCCTCAACCTTGGTTACCTCTGTAAATGGTTTCAAATGTCTTCCTCTTGCAACAAATGGAGCAGCCATCAGAATCATGGCCAAAAATAAGCCAGTCCAAAGCCTTGTCAGCGCCAAATATGATAATTTGACAGTTGATAGGAGATCAGCAAACTACCAACCTTCAATTTGGGACCATGATTTTTTGCAGTCACTGAATAGCAACTATACGGATGAAACATACAAAAGACGAGCAGAAGAGCTGAAGGGAAAAGTGAAGACAGCGATTAAGGATGTAACCGAGCCTCTGGATCAGTTGGAGCTGATAGATAATTTGCAAAGACTTGGATTGGCTTATCATTTTGAGCCTGAGATTCGGAACATATTGCGTAATATCCACAACCATAATAAAGATTATAATTGGAGAAAAGAAAATCTGTATGCAACCTCCCTTGAATTCAGACTACTTAGACAACATGGCTATCCTGTTTCTCAAGAGGTTTTCAGTGGTTTTAAAGACGACAAGGGAGGCTTCATTTGTGATGATTTCAAGGGAATACTGAGCTTGCATGAAGCCTCGTATTACAGCTTAGAAGGAGAAAGCATCATGGAGGAGGCCTGGCAATTCACCAGTAAGCATCTTAAAGAAATGATGATCATCAGCAACAGCAAGGAAGAGGATGTATTTGTAGCAGAACAAGCGAAGCGTGCGCTGGAGCTCCCTCTGCATTGGAAAGTGCCTATGTTAGAGGCAAGGTGGTTCATACACGTTTATGAGAAAAGAGAGGACAAGAACCACCTTTTACTTGAGCTCGCTAAGTTGGAGTTTAACACTTTGCAGGCAATTTACCAGGAAGAACTTAAAGACATTTCAGGGTGGTGGAAGGATACAGGTCTTGGAGAGAAATTGAGCTTTGCGAGGAACAGGTTGGTAGCGTCCTTCTTATGGAGCATGGGGATCGCGTTTGAGCCTCAATTCGCCTACTGCAGGAGAGTGCTCACAATCTCGATAGCCCTAATTACAGTGATTGATGACATTTATGATGTCTATGGAACATTGGATGAACTTGAGCTATTCACTGATGCTGTTGAGAGGTGGGACATCAATTATGCTTTGAAGCACCTTCCGGGCTATATGAAAATGTGTTTTCTTGCGCTTTACAACTTTGTTAATGAATTTGCTTATTACGTTCTCAAACAACAGGATTTTGATATGCTTCTGAGCATTAAAAATGCATGGCTTGGCTTAATACAAGCCTACTTGGTGGAGGCGAAATGGTACCATAGCAAGTACACACCGAAACTGGAAGAATACTTGGAAAATGGATTGGTATCAATAACGGGCCCTTTAATTATAACGATTTCATATCTTTCTGGTACAAATCCAATCATTAAGAAGGAACTGGAATTTCTAGAAAGTAATCCAGGTATAGTTCACTGGTCATCCAAGATTTTCCGTCTGCAAGATGATTTGGGAACTTCATCGGACGAGATACAGAGAGGGGATGTTCCAAAATCAATCCAGTGTTACATGCATGAAACTGGTGCCTCGGAGGAAGTTGCTCGTGAACACATCAAGGATATGATGAGACAGATGTGGAAGAAGGTGAATGCATACACAGCCGATAAAGACTCTCCCTTGACTCGAACAACTACTGAGTTCCTCTTGAATCTTGTGAGAATGTCCCATTTTATGTATCTACATGGAGATGGGCATGGTGTTCAAAACCAAGAGACTATCGATGTCGGTTTTACATTGCTTTTTCAGCCCATTCCCTTGGAGGACAAAGACATGGCTTTCACAGCATCTCCTGGCACCAAAGGCTGA

Note: The cDNA sequence of the chloroplast-targeting peptide is labeled with gray shading.

>STPS-protein

MSSCINPSTLVTSVNGFKCLPLATNGAAIRIMAKNKPVQSLVSAKYDNLTVDRRSANYQPSIWDHDFLQSLNSNYTDETYKRRAEELKGKVKTAIKDVTEPLDQLELIDNLQRLGLAYHFEPEIRNILRNIHNHNKDYNWRKENLYATSLEFRLLRQHGYPVSQEVFSGFKDDKGGFICDDFKGILSLHEASYYSLEGESIMEEAWQFTSKHLKEMMIISNSKEEDVFVAEQAKRALELPLHWKVPMLEARWFIHVYEKREDKNHLLLELAKLEFNTLQAIYQEELKDISGWWKDTGLGEKLSFARNRLVASFLWSMGIAFEPQFAYCRRVLTISIALITVIDDIYDVYGTLDELELFTDAVERWDINYALKHLPGYMKMCFLALYNFVNEFAYYVLKQQDFDMLLSIKNAWLGLIQAYLVEAKWYHSKYTPKLEEYLENGLVSITGPLIITISYLSGTNPIIKKELEFLESNPGIVHWSSKIFRLQDDLGTSSDEIQRGDVPKSIQCYMHETGASEEVAREHIKDMMRQMWKKVNAYTADKDSPLTRTTTEFLLNLVRMSHFMYLHGDGHGVQNQETIDVGFTLLFQPIPLEDKDMAFTASPGTKG*

Note: the conserved domain in the protein sequence is labeled with turquoise shading.
