# Supplementary material for: Transcriptomic and metabolomic analyses provide insight into the volatile compounds of citrus leaves and flowers
Source: BMC Plant Biol. 2020 Jan 6;20:7. doi: 10.1186/s12870-019-2222-z (PMC6945444; doi:10.1186/s12870-019-2222-z)
Supplement: Supplementary file 20 — Additional file 20: Figure S9. A: alignment of deduced amino acid sequences between STPS and four D-limonene synthase genes. The amino acid sequences of KU746814, AB110637, AB266584 and Cs3g04360 were obtained from the NCBI website and the genome sequence of Valencia sweet orange. B: sabinene content and relative expression level of STPS. C: purification of His-tagged STPS protein analyzed by SDS-PAGE in E.coli and Western blot analysis. M: Protein Ladder (#SM0671); 1: STPS-tr flow-through of the lysate from a Ni-NTA His-Bind Resin column; 2, 3: eluate of STPS; 4: STPS-fl flow-through of the lysate from a Ni-NTA His-Bind Resin column; 5, 6: eluate of STPS-fl. D: Identification of recombinant STPS-tr and STPS-fl by Western blot analysis. [file 12870_2019_2222_MOESM20_ESM.pdf]

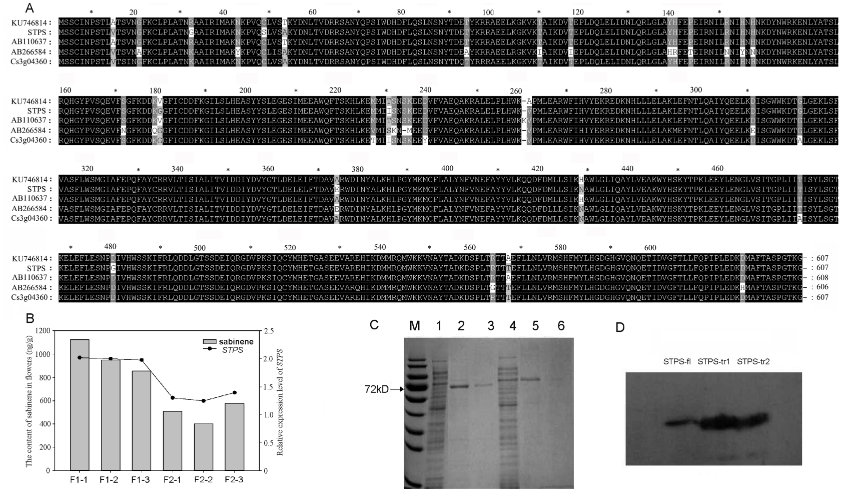
A
KU746814 :
STPS :
AB110637 :
AB266584 :
Cs3g04360 :
: 607
: 607
: 608
: 606
: 607
B
sabinene
STPS
The content of sabinene in flowers (ng/g)
Relative expression level of STPS
F1-1
F1-2
F1-3
F2-1
F2-2
F2-3
C
M
1
2
3
4
5
6
72kD
D
STPS-fl
STPS-tr1
STPS-tr2
